# Exosome and BCR-ABL mediated molecular alterations in endothelial cells in chronic myeloid leukemia: identification of seven genes and their regulatory network

**DOI:** 10.7717/peerj.20371

**Published:** 2025-12-17

**Authors:** Zhenglei Shen, Honghua Cao, Yeying Zhou, Wenwen Mao, Kunmei Liu, Jingying Zhu, Ming He, Yunru Mao, Ni Luo, Lei Feng, Heng Le, Liying Song, HuaXian Li, Yasar Mehmood Yousafzai, Asad Zia, Xuezhong Gu, Shiwen Zhang

**Affiliations:** 1 Department of Hematology, The Third Affiliated Hospital of Kunming Medical University, Kunming, China; 2 Department of Hematology, Longhua District People’s Hospital, Shenzhen, China; 3 Department of Geriatics, The Second Hospital of Kunming, Kunming, China; 4 NO.1 Head and Neck External Department, The Third Affiliated Hospital of Kunming Medical University, Kunming, China; 5 No.10 Middle School, Kunming, China; 6 Department of Hematology, The Third Affiliated Hospital of Kunming Medical University, Kunming, China; 7 Institute of Pathology and Diagnostic Medicine Khyber Medical University, Peshawar, Pakistan; 8 Molecular Biologist Public Health Reference Laboratory, Peshawar, Pakistan; 9 Department of Hematology, The First People Hospital in Yunnan Province, Kunming, China

**Keywords:** Chronic myeloid leukemia (CML), Human umbilical vein endothelial cells(HUVECs), K562 cell line, Exosomes, Biomarkers

## Abstract

**Background:**

Chronic myeloid leukemia (CML) progression relies on dynamic crosstalk between leukemic cells and vascular niches. Here, we investigate how exosomes and BCR/ABL overexpression influence endothelial functions, aiming to identify key mediators of leukemia-induced microenvironmental remodeling as potential therapeutic targets.

**Methods:**

Human umbilical vein endothelial cells (HUVECs) were cultured and divided into four groups: control (Z), treated with K562-derived exosomes (Zexo), BCR-ABL-overexpressing (ZBA), and BCR-ABL-overexpressing with exosome treatment (ZBAexo). Transcriptomic profiling was performed to identify DEGs, followed by functional enrichment and protein–protein interaction network analyses. Gene Set Enrichment Analysis (GSEA) was applied to uncover associated biological pathways.

**Results:**

Seven specificized expressed genes in ZBAexo group were identified: *CAMK2B*, *CDC25C*, *SV2A*, *MND1*, *CDC20*, *CLSPN*, and *GRM1*. These genes are involved in cell cycle, DNA replication, and cell adhesion pathways and show significant correlation with the BCR/ABL fusion gene. Expression of these genes was strongly associated with BCR-ABL. Network analysis revealed the potential regulatory roles of transcription factors *CREB1* and *NFKB1*. A competing endogenous RNA (ceRNA) network involving miRNAs (*e.g.*, miR-16-5p, miR-126-5p) and lncRNAs (*e.g.*, AC008124.1, AC064799.2, AGAP11) potentially modulates their expression.

**Conclusion:**

This study identifies seven novel candidate biomarkers dysregulated in endothelial cells under combined BCR-ABL and exosomal stimulation, shedding light on the molecular crosstalk between leukemic cells and the vascular niche.

## Introduction

Chronic myeloid leukemia (CML) is a relatively uncommon but clinically emblematic malignancy, representing nearly 15% of all leukemias with an annual incidence of 1.6∼2.0 per 100,000 worldwide (Jabbour & Kantarjian, 2022). CML is initiated by the Philadelphia chromosome, which results from the t (9;22) translocation and generates the BCR-ABL1 fusion oncogene. This constitutively active tyrosine kinase activates downstream signaling pathways such as PI3K/AKT, RAS/MAPK, JAK/STAT, and NF-κB, promoting uncontrolled proliferation and survival of myeloid cells. BCR-ABL1 also induces genomic instability through increased reactive oxygen species (ROS), leading to DNA damage and error-prone repair mechanisms like non-homologous end joining, which contribute to disease progression (Braun, Eide & Druker, 2020; Sun et al., 2024).

CML has experienced a significant increase in survival rates due to the introduction of tyrosine kinase inhibitors (TKIs). The introduction of imatinib and successive TKIs has shifted the natural history of the disease: 5-year overall survival now approaching 90–95% and 10-year survival still exceeding 80%. However, TKI resistance remains an unresolved clinical reality. First-line imatinib fails in 10–15% of patients within a decade, and even second-generation TKIs exhibit a cumulative resistance rate of up to 10%. In Chinese, imatinib was associated with a significantly higher annual risk of treatment failure compared to nilotinib (0.199 *vs.* 0.041). Patients who experienced TKI failure were more likely to be younger (median age: 38.6 years), present with progressive disease (44.3%) (Zhang et al., 2022).

The development of resistance is often linked to BCR/ABL-dependent and -independent mechanisms. BCR/ABL-dependent resistance is primarily associated with point mutations within the kinase domain of the BCR/ABL fusion protein, which can modify the drug-binding site or alter its conformation, thereby diminishing the affinity of TKIs for their target (Lyczek et al., 2021). Additionally, BCR/ABL-independent resistance mechanisms include the efflux of drugs *via* ATP-binding cassette transporters, such as P-glycoprotein, which can lead to reduced intracellular concentrations of TKIs (Howlader et al., 2023). These mechanisms collectively limit the depth of molecular response and hinder treatment-free remission in some patients. Endothelial cells (ECs) play a critical role in the survival, proliferation, and quiescence of leukemic stem and progenitor cells in CML. These cells not only create a supportive microenvironment for leukemic cells but also participate in angiogenesis and adhesion processes, which contribute to disease severity and therapeutic resistance. In the context of TKI resistance in CML, ECs are implicated through several mechanisms. One notable mechanism is the endothelial-to-mesenchymal transition (EndMT), a process associated with various vascular pathologies, which may also play a role in TKI resistance. Research has shown that dasatinib can induce EndMT in human vascular endothelial cells, characterized by a decrease in endothelial markers and an upregulation of mesenchymal markers (Alkebsi et al., 2021). Moreover, the interaction between CML cells and the vascular microenvironment significantly influences TKI resistance. ECs within the bone marrow niche serve as sanctuary sites for leukemic cells, protecting them from the cytotoxic effects of TKIs. The E-selectin—SCL/TAL1—CD44 axis has been identified as a critical pathway mediating this interaction, presenting potential therapeutic targets for disrupting these protective niches and overcoming TKI resistance (Godavarthy et al., 2020).

Extracellular vesicles (EVs)—and exosomes in particular—have emerged as dominant vectors of EC-driven reprogramming. Studies have demonstrated that exosomes secreted by leukemia cells can reprogram the bone marrow microenvironment to facilitate disease progression. Recent reviews highlight that CML-derived EVs shuttle not only miRNAs but also long non-coding RNAs, cytokines, and even BCR-ABL1 mRNA, thereby propagating pro-survival signals and transferring drug resistance to bystander cells (Bernardi et al., 2023; Zheng, Wang & Ge, 2025). For example, Gao et al. (2019) reported that exosomal miR-320 from leukemia cells significantly inhibits osteogenesis in bone marrow mesenchymal stromal cells (BMMSCs), both *in vivo* and *in vitro*, resulting in enhanced leukemia cell proliferation. Additionally, Wan et al. (2019) found that CML-derived exosomes containing miR-92a-3p suppress adipogenesis in adipose-derived mesenchymal stem cells, contributing to cancer-associated cachexia and poor treatment tolerance.

The impact of exosomes on angiogenesis, a key factor in leukemia progression, has also been elucidated. Exosomes from K562 CML cells were found to promote angiogenesis in a Src-dependent manner, suggesting a potential therapeutic target (Corrado et al., 2014). Furthermore, the crosstalk between CML cells and human bone marrow stromal cells *via* exosomes triggers an interleukin 8-dependent survival of leukemia cells, highlighting their involvement in chemoresistance (Corrado et al., 2014). In relation to drug resistance, Hrdinova et al. (2021) characterized exosomes released by imatinib-resistant K562 cells could increase the survival of imatinib-sensitive cells in the presence of the drug, indicating a role of exosomes in conferring resistance. Additionally, Chen et al. (2022) reported that human umbilical cord mesenchymal stem cell-derived exosomes promote imatinib-induced apoptosis in K562-R cells *via* a miR-145a-5p/USP6/GLS1 axis, highlighting the potential of exosome-based therapies to overcome chemoresistance.

These findings underscore the significance of exosomes in leukemia biology and suggest that targeting exosome pathways may provide novel therapeutic strategies for the treatment of leukemia and overcoming drug resistance. In this research, we aimed to employ whole transcriptome sequencing to identify differentially expressed genes resulting from dual stimulation by K562-derived exosomes and the Bcr/Abl fusion gene. This approach was designed to uncover potential biomarkers that could assist in the diagnosis and guide personalized therapeutic strategies for CML.

## Materials and Methods

### Preparation of exosomes

Exosomes derived from K562 cells were isolated using the Ultracentrifugation Method (An et al., 2018). The K562 cell line was cultured to the logarithmic growth phase, and the cell culture supernatant was collected. The supernatant was first centrifuged at 2,000 g for 10 min and then at 10,000 g for 30 min at 4 °C to remove cell debris and large particulate matter. The resulting supernatant was transferred to Ultra-Clear tubes (Beckman Coulter, Indianapolis, IN, USA), diluted with PBS, and subjected to ultracentrifugation at 100,000 g for 120 min at 4 °C using a Beckman Optima XL-70 ultracentrifuge. To effectively eliminate serum protein contamination, five cycles of ultracentrifugation were conducted. Nano-Flow Cytometry (NanoFCM U30) were subsequently employed to measure the size of the exosomes for further analysis. The exosome concentration was determined to be 1.09  ×   10^1^^1^ particles/mL and used for subsequent experiments.

### Western blot analysis for exosome characterization

To molecularly validate the successful isolation and purity of the exosomes, Western blot analysis was performed for the detection of specific exosomal markers and the absence of contaminants. Whole cell lysate (WCL) and purified exosome samples were lysed using RIPA buffer supplemented with protease inhibitors. Subsequently, 20 µg of exosomal protein and 10 µg of WCL protein were separated by SDS-PAGE on 10% gels and transferred to PVDF membranes. The membranes were incubated overnight at 4 °C with primary antibodies against TSG101 (1:1000, #ab125011; Abcam, Cambridge, UK), CD63 (1:1000, #ab134045; Abcam, Cambridge, UK), HSP70 (1:1000, #ab181606; Abcam, Cambridge, UK), and Calnexin (1:500, #10427-2; Rosemont, IL, USA). Following washes, HRP-conjugated secondary antibodies were applied for 1 h at room temperature. Protein bands were visualized using an ECL substrate on a chemiluminescence imaging system.

### Cells culture

**Figure 1:**
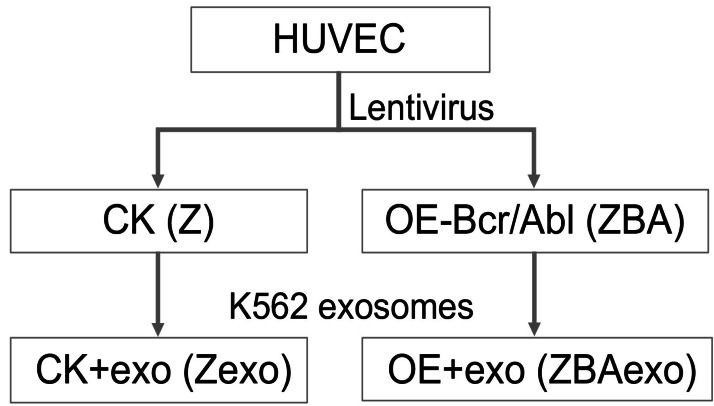
Cell line model construction and grouping.

In the study, K562 cells or human umbilical vein endothelial cells (HUVEC) were purchased from ATCC and cultured in RPMI-1640 medium and at 37 °C, 5% CO_2_ and 70–80% humidity. The HUVEC cells were divided into four groups (Fig. 1): HUVEC cells (Z), HUVEC cells co-cultured with K562 exosomes (Zexo), BCR-ABL overexpressed HUVEC cells (ZBA), BCR-ABL overexpressed HUVEC cells co-cultured with K562 exosomes (ZBAexo). A lentiviral vector (pHS-AVC) was constructed to overexpress BCR-ABL, and viral particles were produced at a titer of 2.39  × 10^8^ TU/mL. HUVECs were then transduced and stably selected with puromycin to generate the BCR-ABL-overexpressing cell line (ZBA group). Cells were collected and immediately frozen in liquid nitrogen, then stored at −80 °C for RNA extraction.

### Cell function assay

To evaluate the functional phenotypes of endothelial cells, we compared control HUVEC cells (Z group) and BCR-ABL-overexpressing HUVEC cells (ZBA group).

Cell proliferation was assessed using the CCK8 assay. Cells in Z and ZBA group were seeded at 4,000 cells per well in 96-well plates. Cell viability was measured after 24, 48, 72, and 96 h of culture. At each time point, 10 µL of CCK8 solution was added to each well and incubated for 3 h at 37 °C. Absorbance at 450 nm was determined using a microplate reader (PerkinElmer, Waltham, MA, USA), and data were analyzed to generate growth curves.

Cell cycle distribution was analyzed by flow cytometry. Cells were harvested, washed with PBS, and stained with a DNA dye in the dark at room temperature for 15 min. Samples were analyzed using an analytical flow cytometer (CytExpert, Beckman, Brea, CA, USA).

Apoptosis analysis: cells were detached with trypsin (GIBCO, Cat# 25200072), washed with PBS, and resuspended in binding buffer containing Annexin V-Alexa Fluor 647 and 10 µL PI (Yisheng Biotechnology, Cat# 40304ES20). Samples were incubated for 15 min at room temperature in the dark. Apoptotic cells were detected within one hour by flow cytometry (CytExpert, Beckman, Brea, CA, USA).

Colony formation and angiogenic capacity were assessed using a Matrigel-based tube formation assay. Cells were resuspended at a density of 3 × 10^5^ cells/mL and seeded onto growth factor-reduced Matrigel (Cat# 356234; BD, Franklin Lakes, NJ, USA). Plates were incubated at 37 °C, and tubular network formation was monitored starting 3 h post-seeding. Images were captured at defined time points to document morphological changes and vessel-like structure development.

### Transcriptome sequencing

Total RNA was extracted from cells of the four groups using the TRIzol method followed by the Qiagen RNA extraction kit (Qiagen, Hilden, Germany). Each group included three biological replicates. The strand-specific cDNA libraries were constructed according to the TruSeq Stranded Total RNA Library Prep protocol (Illumina, San Diego, CA, USA). Libraries were sequenced on the Illumina HiSeq 2500 platform in paired-end mode (150 bp read length). A total of 234.14 Gb clean data were generated from 12 samples, with each sample yielding at least 16.22 Gb of clean data. The Q30 score was higher than 89.23% for each sample. Clean reads were aligned to the human reference genome GRCh38 (http://ftp.ensembl.org/pub/release-101/fasta/homo_sapiens/dna/) using HISAT2 (Kim, Langmead & Salzberg, 2015). The alignment efficiency ranges from 95.42% to 96.56%, yielding an average of 127.3 ± 11.8 million reads per sample. Transcriptome assembly were performed using StringTie (Kovaka et al., 2019). Concurrently, small RNA libraries were prepared from the same RNA samples and sequenced on the HiSeq 2500 platform, generating 189.97 million clean reads (average of 15.83 million reads per sample).

### Differential gene expression analysis

Principal component analysis (PCA) was performed to assess the similarity of gene expression profiles within groups and the separation between groups. Bioinformatic analyses were conducted using a log2-transformed, normalized HTSeq-FPKM matrix (log2(FPKM+1)). Differential expression analysis between sample groups was carried out using the R package DESeq2. Genes with an adjusted *p*-value (padj) < 0.05 and —log2 fold change—≥ 1 were considered significantly differentially expressed. This criterion was applied to identify differentially expressed mRNAs, lncRNAs, and miRNAs. For miRNA, only those with TPM greater than 10 were retained for differential expression testing.

Venn diagrams were generated using the R package VennDiagram to identify ZBAexo-specific differentially expressed genes (ZBAexo-sDEGs). Gene Ontology (GO) and Kyoto Encyclopedia of Genes and Genomes (KEGG) pathway analysis of ZBAexo-sDEGs was performed using the R package clusterProfiler.

### Analysis of tumor-related signaling pathways involving DEGs

Gene sets related to key cancer hallmarks were obtained from the Molecular Signatures Database (MSigDB; https://www.gsea-msigdb.org/gsea/msigdb/), including 125 cell cycle–associated genes, 680 apoptosis–related genes, 36 angiogenesis–related genes, and 156 cell adhesion–related genes. Single-sample gene set enrichment analysis (ssGSEA), implemented in the R package GSVA, was applied to calculate pathway activity scores for apoptosis, angiogenesis, and cell adhesion across the four sample groups. Differences in pathway enrichment scores among groups were assessed using the Kruskal–Wallis test. To identify specific signaling pathways associated with ZBAexo-sDEGs, an intersection analysis was performed between ZBAexo-sDEGs and the cancer hallmark gene sets downloaded from MSigDB, with overlapping genes identified using Venn diagram analysis.

### Identification of hub genes through protein-protein interaction network analysis

The PPI network of ZBAexo-sDEGs was constructed using the STRING database (https://string-db.org/). A minimum interaction score of above 0.4 was considered to be significant. The resulting PPI network was imported into Cytoscape and analyzed using the cytoHubba plugin to identify hub genes based on topological features. We employed ten algorithms to rank the genes nodes by their network importance, including Maximum Neighborhood Component (MNC), Maximal Clique Centrality (MCC), Edge Percolated Component (EPC), BottleNeck, Radiality, Eccentricity, Density of Maximum Neighborhood Component (DMNC), Betweenness, Degree, and Closeness. For every algorithm, the 30 highest-ranking genes were subjected to intersection analysis using the UpSetR package in R. To evaluate the pairwise co-expression patterns among identified hub genes, Spearman’s rank correlation coefficients were calculated based on their expression levels through the corrplot package in R. Furthermore, the association between hub gene expression and pathway activity scores (apoptosis, angiogenesis, and cell adhesion, derived from ssGSEA) was evaluated using R package psych. Results were visualized as lollipop plots to illustrate significant correlations.

### Potential drug identification and construction of regulatory networks

To identify potential therapeutic agents targeting the hub genes, drug-gene interaction predictions were performed using the Drug Gene Interaction Database (DGIdb; https://dgidb.org/). Putative transcription factors (TFs) regulating the expression of these hub genes were inferred through the NetworkAnalyst platform (https://www.networkanalyst.ca/). To construct a competing endogenous RNA (ceRNA) network, potential interactions between the hub genes and differentially expressed miRNAs and lncRNAs were predicted using the starBase database (https://starbase.sysu.edu.cn/). All predicted interactions—including drug-hub gene, TF-hub gene, lncRNA-miRNA, and miRNA-hub gene relationships—were integrated and visualized as a comprehensive regulatory network using Cytoscape.

### Quantitative Real-Time PCR

Genomic DNA was removed using a gDNA Wiper Mix (Vazyme Biotech Co., Ltd., Beijing, China) at 42 °C for 2 min. Reverse transcription was performed with the HiScript^®^ III 1st Strand cDNA Synthesis Kit (Vazyme Biotech Co., Ltd.) at 37 °C for 15 min, followed by 85 °C for 5 s. qRT-PCR was conducted using the UltraSYBR Mixture (Kangwei Century Biotech Co., Ltd., Beijing, China) on the Thermo Fisher Scientific 7500 Real-Time PCR System The reaction system (20 µL) contained 10 µL of 2 × SYBR Green, 0.4 µL of each primer (10 µM), two µL of cDNA template, and 7.2 µL of RNAase-free H_2_O. The PCR program was set as follows: initial denaturation at 95 °C for 30 s, followed by 50 cycles of denaturation at 95 °C for 5 s, and annealing/extension at 58 °C for 30 s. GAPDH was used as the internal reference gene. Each experiment was performed with three biological replicates. Primers used were as follows: BCR-ABL-F (CGGAATGCTGTGGACAGTCT), BCR-ABL-R (GGGAGCAGCAGAAGAAGTGT), GAPDH-F (GAGTCAACGGATTTGGTCGT), and GAPDH-R (GACAAGCTTCCCGTTCTCAG). Data were analyzed using the ΔΔCt method, with positive and negative controls included to ensure amplification accuracy. Each experiment was performed with three biological replicates, and each qRT-PCR reaction was run in triplicate to ensure data reliability and reproducibility.

### Statistical analysis

The statistical power, calculated using RNASeqPower with three biological replicates and a significance level (α) of 0.1, is 0.87. In the study, Correlation analysis was performed by Spearman’s correlation. *P* < 0.05 was deemed statistically significant in all situations. All statistical analyses were performed using R software (version 4.2.0).

## Results

### Overexpression of BCR/ABL leads to malignant proliferation of HUVEC cells

We initially utilized lentiviral transduction to establish a HUVEC cell line overexpressing BCR-ABL (ZBA group). Quantitative reverse transcription polymerase chain reaction (qRT-PCR) was employed to assess BCR/ABL expression, revealing that the expression levels in the stably transfected HUVEC cells were elevated to 23-fold relative to the control group (Z group) (Fig. S1A). Cell Counting Kit-8 (CCK8) assays demonstrated a significant enhanced proliferation in the ZBA group compared to the Z group (Fig. S1B). Cell cycle analysis indicated no significant difference in the proportion of cells in the G2/M phases; cells in the G0/G1 phase were significantly reduced in ZBA group. However, the proportion of cells in the S phase was significantly increased in the ZBA group (*P* < 0.001) (Fig. S1C). Furthermore, apoptosis rates in the ZBA group were significantly reduced (*P* < 0.05) (Fig. S1H), while angiogenesis was enhanced (*P* < 0.01) (Figs. S1D & S1F), and the number of cell colonies was markedly increased (*P* < 0.01) (Figs. S1E & S1G).

### Exome preparation and validation

To investigate the effects of K562-derived exosomes on endothelial cells, exosomes were first isolated from K562 cell culture supernatants and subsequently co-cultured with cells of the ZBA or Z group. Nano-Flow Cytometry was performed to characterize the isolated exosomes (Fig. S2A). The results showed that the particle size distribution ranged from 42.4 to 106.1 nm, with a median diameter of 54.25 nm and a mean diameter of 57.16 nm (SD = 10.62 nm). Western blot analysis was performed to validate the successful isolation and purity of the exosomes. As shown in Fig. S2B, the exosome fractions showed strong positive signals for the canonical exosomal markers TSG101 (45 kDa), CD63 (26 kDa), and HSP70 (70 kDa), confirming the enrichment of exosome-origin vesicles. In contrast, the endoplasmic reticulum protein calnexin (90 kDa), which was abundantly detected in the whole-cell lysate (WCL) control, was absent in the exosome samples. This marker expression profile confirms the successful preparation of highly enriched exosomes suitable for downstream functional analyses.

### Identification of DEGs specifically expressed in the ZBAexo group

The Z, Zexo, ZBA and ZBAexo groups were subjected to high-throughput RNA sequencing. To evaluate the reproducibility of the sequencing data, principal component analysis (PCA) was performed. Overall, the degree of sample clustering within groups is higher than that between groups (Fig. S3). A total of 38 DEGs were identified between the Zexo and Z groups (Zexo-DEGs), comprising 23 upregulated genes and 15 downregulated genes (Figs. S4A–S4B). A sum of 484 DEGs were obtained between ZBA and Z groups, including 354 upregulated genes and 130 downregulated genes, termed as ZBA-DEGs (Figs. S4C–S4D). When comprising the ZBAexo and Z groups, 542 DEGs were identified, termed as ZBAexo-DEGs, consisting of 392 upregulated genes and 150 downregulated genes (Figs. S4E–S4F).

**Figure 2:**
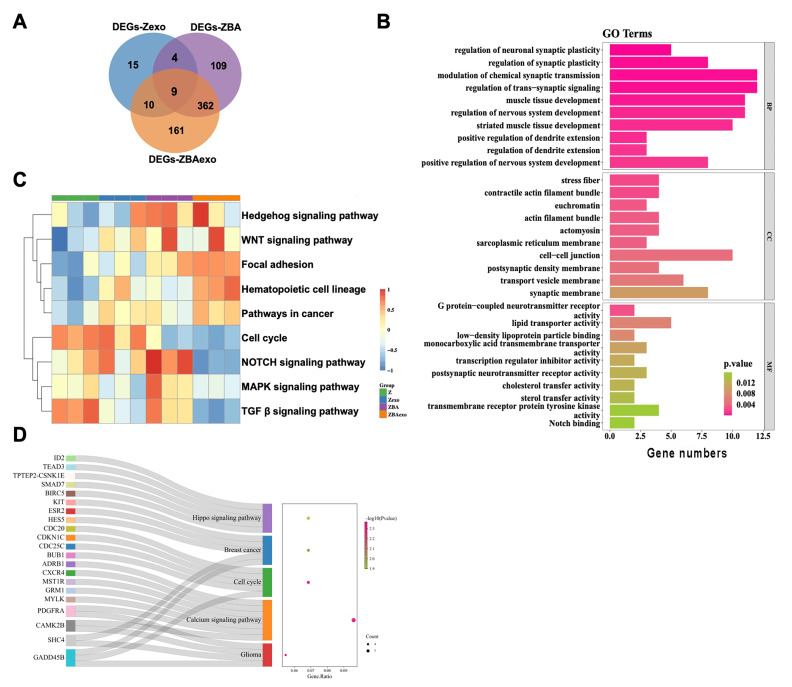
Identification and functional analysis of differentially expressed genes in the ZBAexo group. (A) Venn diagram of DEGs in ZBAexo *vs* Z, ZBA *vs* Z, and Zexo *vs* Z. (B) GO enrichment analysis of ZBAexo-specific DEGs (C) Gene set variation analysis of ZBAexo-specific DEGs (D) KEGG enrichment analysis of ZBAexo-specific DEGs.

To identify DEGs specifically associated with the combined effect of K562-derived exosomes and BCR-ABL overexpression, we performed an intersection analysis of Zexo-DEGs, ZBA-DEGs, and ZBAexo-DEGs, resulting in a set of 161 unique DEGs present only in the ZBAexo group (Fig. 2A). Functional enrichment analysis of these 161 ZBAexo specific DEGs revealed significant enrichment in biological processes such as “modulation of chemical synaptic transmission”, “cell–cell junction”, and “Notch binding” (Fig. 2B). Additionally, five Kyoto Encyclopedia of Genes and Genomes (KEGG) pathways were significantly enriched, including “cell cycle”, “calcium signaling pathway”, “Hippo signaling pathway”, “breast cancer”, and “glioma” (Fig. 2C). To further investigate expression differences among all groups, a total of 64 signaling pathways were enriched through Gene Set Variation Analysis (GSVA). The GSVA results indicated that the KEGG pathways for “cell cycle”, “Notch signaling pathway”, “MAPK signaling pathway”, and “TGF-beta signaling pathway” were significantly upregulated in the ZBAexo group compared to the other groups (Fig. 2D).

### Screening and verifying diagnostic biomarkers

**Figure 3:**
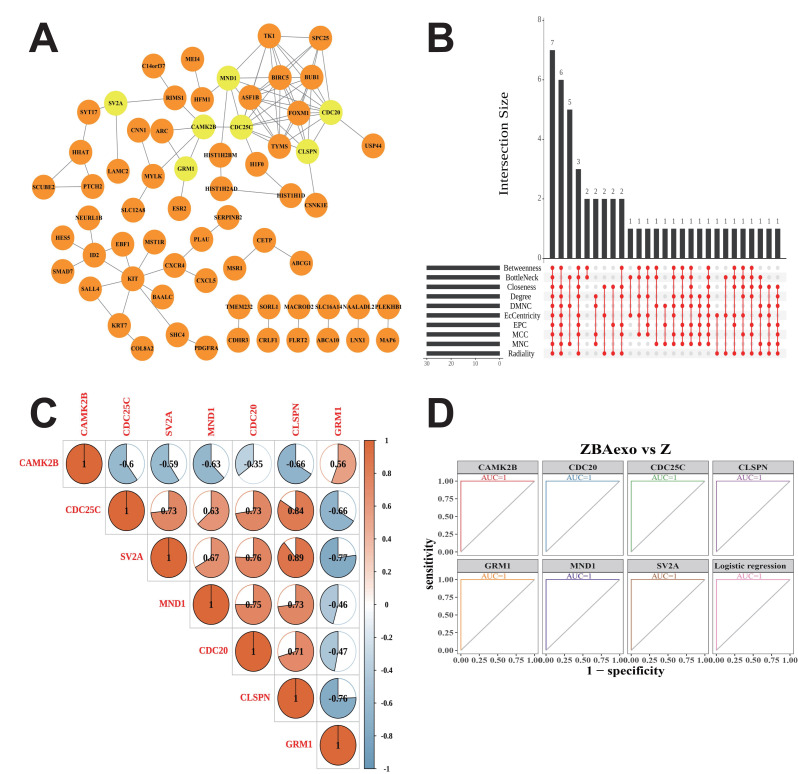
Protein-protein interaction analysis and identification of hub genes. (A) Protein-protein interaction network for 161 candidate genes; (B) UpSet plot of top 30 genes selected by 10 algorithms; (C) Correlation analysis of seven hub genes; (D) AUC analysis of seven genes differentiating ZBAexo and Z groups.

The top 30 genes in 10 ranked algorithms were figured out, and the intersections between the ten algorithms were considered as potential diagnostic biomarkers of CML (Fig. 3A). Ultimately, seven diagnostic biomarkers were obtained, which were *CAMK2B*, *CDC25C*, *SV2A*, *MND1*, *CDC20*, *CLSPN*, and *GRM1* respectively (Fig. 3B). Figure 3C illustrates the correlation among these biomarkers, revealing a strong positive association between *CDC25C* and *CLSPN*, as well as between *SV2A* and *CLSPN*, with correlation coefficients exceeding 0.8. Based on those biomarkers, a logistic regression model was constructed. Additionally, we performed receiver operating characteristic (ROC) analysis to assess the diagnostic significance of the identified biomarkers. The area under the curve (AUC) for all models was 1, indicating the high discriminatory power of the biomarker panel in this controlled *in vitro* system, where the combined BCR-ABL overexpression and exosomal stimulation induces a robust and distinct transcriptional signature (Fig. 3D).

### Analyses of biological processes and core pathways enriched for biomarkers

**Figure 4:**
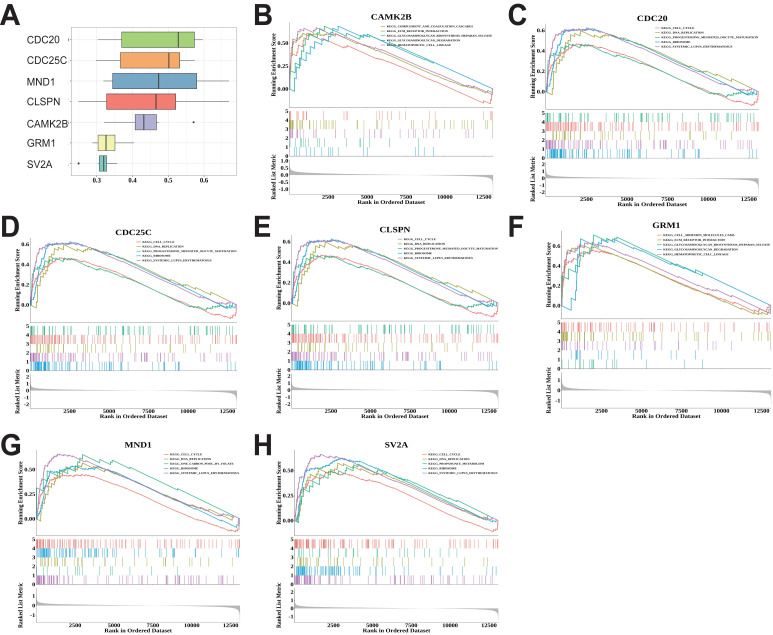
Functional analysis of seven candidate genes. (A) Functional similarity analysis of seven candidate genes; (B)–(H) Gene Set Enrichment Analysis for seven candidate genes.

Functional similarity of the seven biomarkers was analyzed. The *CDC20* had higher functional similarity and the others had low functional similarity, which indicated the seven biomarkers were highly representative (Fig. 4A). To elucidate the biological pathways associated with these biomarkers, we performed Gene Set Enrichment Analysis (GSEA). The GSEA results revealed the top five enriched pathways for each biomarker (Figs. 4B–4H). Among them, *CDC20*, *CDC25C*, *CLSPN*, *MND1* and *SV2A* were enriched cell cycle, DNA Replication signaling pathway. *CAMK2B* was enriched ECM receptor interaction, complement and coagulation cascades and cell lineage signaling pathway. *GRM1* was enriched ECM receptor interaction and cell adhesion signaling pathways.

### The relationship between ZBAexo-sDEGs and core signaling pathways genes

**Figure 5:**
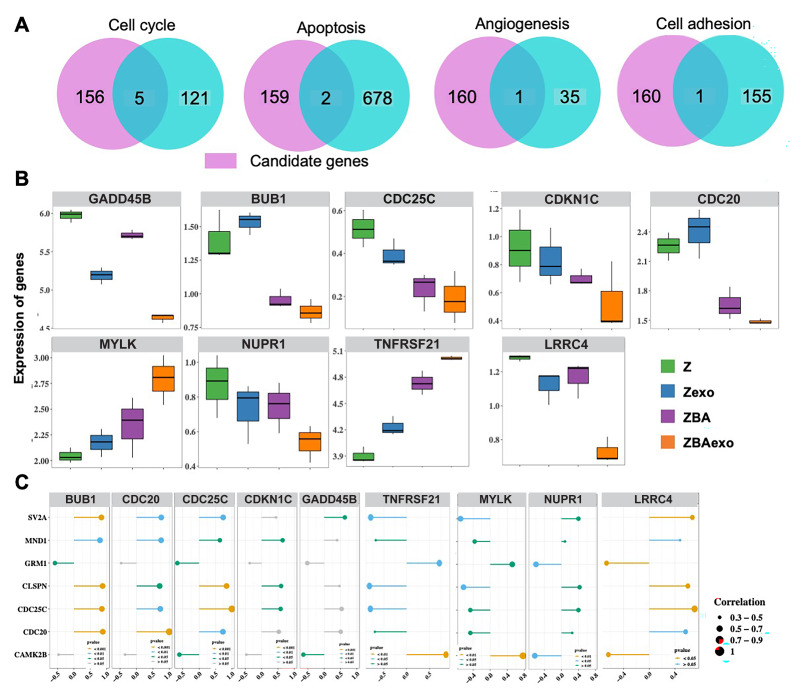
Identification of cell function-related genes in ZBAexo-Specific expressed genes. (A) Selection of genes involved in cell cycle, apoptosis, angiogenesis, and cell adhesion regulation among ZBAexo-specific expressed genes. (B) Box plots showing the expression levels of cell cycle, apoptosis, angiogenesis, and cell adhesion regulation-related genes uniquely present in ZBAexo across four groups of samples. (C) Lollipop plots illustrating the expression of cell cycle, apoptosis, angiogenesis, and cell adhesion regulation-related genes uniquely present in ZBAexo in relation to hub genes.

The results of GSEA showed that the seven biomarkers were significantly enriched in pathways related to the cell cycle, apoptosis, angiogenesis, and cell adhesion signaling. To further investigate the relationship between the ZBAexo-specific DEGs and these key signaling pathways, we identified five overlapping genes associated with the cell cycle (*GADD45B, BUB1, CDC25C, CDKN1C*, and *CDC20*), two overlapping genes linked to apoptosis (*MYLK* and *NUPR1*), one overlapping gene related to angiogenesis (*TNFRSF21*), and one overlapping gene associated with cell adhesion (*LRRC4*) (Fig. 5A). Interestingly, both *CDC25C* and *CDC20* were present among the nine overlapping genes and the seven biomarkers simultaneously. The expression of these nine genes were shown in Fig. 5B. Among them, *MYLK* and *TNFRSF21* exhibited significantly higher expression in the ZBAexo group compared to the Z group (*p* < 0.05), while the expression of the remaining genes was inversely related. Subsequently, we assessed the regulatory relationships between these nine genes and the seven biomarkers using Spearman correlation analysis based on expression levels (Fig. 5C). Thereinto, *BUB1* and biomarkers were highly positive associated, *MYLK* were highly negative associated. Above them, the angiogenesis and cell adhesion score (ssGSEA score) were significantly different among the four groups. The relationship between ssGSEA scores (apoptosis, angiogenesis and cell adhesion) and seven biomarkers were shown in Fig. S5.

### CeRNA and TF network construction

**Figure 6:**
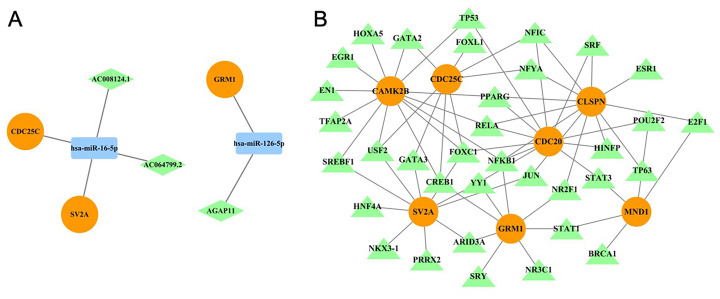
Regulatory network analysis of seven hub genes. (A) MicroRNA regulatory network analysis; (B) Transcription factor regulatory network analysis.

Long non-coding RNA (LncRNA) can enhance the expression level of miRNA target genes by competitively binding to miRNAs. We constructed a competing endogenous RNA (ceRNA) network based on the expression data of miRNAs, lncRNAs, and mRNAs (Fig. S6, Table S1). The interactions between lncRNAs and miRNAs, as well as between miRNAs and mRNAs, were predicted using the ‘starBase’ database. In this network, both *CDC25C* and *SV2A* were regulated by *has-miR-16-5p*, which in turn was regulated by lncRNAs *AC008124.1* and *AC064799.2*. Additionally, another subnetwork consisting of *GRM1*, *has-miR-126-5p*, and *AGAP11* was identified. These findings suggest that the ceRNA network may play a crucial role in chronic myeloid leukemia (CML) by modulating the expression of *CAMK2B*, *CDC25C*, *SV2A*, and *GRM1*. Furthermore, transcription factors (TFs) associated with the seven biomarkers were predicted using the ‘NetworkAnalyst’ database, and the TF-mRNA interaction network was visualized with Cytoscape software (Fig. 6). This analysis identified 35 predicted TFs. Notably, the potential target genes of *CREB1* overlapped with four biomarkers: *CAMK2B*, *SV2A*, *CDC25C*, and *GRM1*. Additionally, the potential target genes of *NFKB1* overlapped with five biomarkers: *CAMK2B*, *SV2A*, *CDC20*, *CLSPN*, and *GRM1*.

### BCR/ABL fusion genes and drug prediction

**Figure 7:**
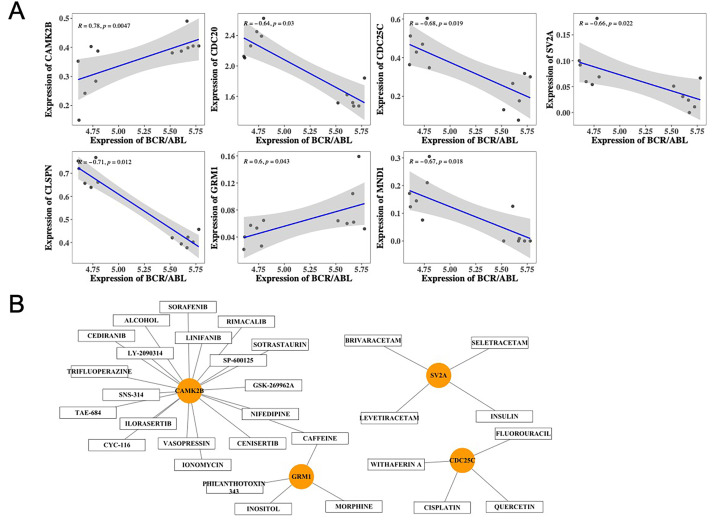
Prediction analysis of small molecule drugs for seven hub genes. (A) Correlation analysis between the expression of seven hub genes and BCR-ABL; (B) Analysis of small molecule drugs corresponding to hub genes.

In this study, we analyzed the correlation between the BCR/ABL fusion gene and the seven biomarkers using Spearman correlation coefficients. Our findings revealed a significant positive correlation between *GRM1* and *CAMK2B* with the BCR/ABL fusion genes. Conversely, *CLSPN*, *CDC25C*, *MND1*, *SV2A*, and *CDC20* exhibited significant negative correlations with BCR/ABL fusion genes (—cor—>0.6, *p* < 0.05) (Fig. 7A). We submitted the biomarkers to the DGidb database to select for small molecule compounds, which could be used for CML management. The small molecule compounds of *CAMK2B*, *CDC25C*, *SV2A* and *GRM1* were predicted, then the results were visualized by Cytoscape (Fig. 7B, Table S2). In total, 30 drugs were predicted to interact with the biomarkers, with caffeine identified as a common predicted compound for both *CAMK2B* and *GRM1*. These results demonstrated comprehensively that the seven biomarkers had potential as diagnostic and therapeutic targets for CML.

## Discussion

The bone marrow microenvironment plays a pivotal role in the pathogenesis and therapeutic resistance of chronic myeloid leukemia (CML), with endothelial cells (ECs) increasingly recognized as key components of the vascular niche that support leukemic stem cell survival and immune evasion. While tyrosine kinase inhibitors (TKIs) have dramatically improved clinical outcomes, intrinsic or acquired resistance remains a significant challenge (Hrdinova et al., 2021), often linked to microenvironment-mediated protective mechanisms (Shammas et al., 2025). Emerging evidence highlights exosomes—nanosized extracellular vesicles secreted by tumor cells—as critical mediators of intercellular crosstalk that reprogram stromal cells to foster a pro-leukemic niche (Bernardi et al., 2023). In fact, recent advances underscore the multifaceted roles of exosomes in myeloid leukemia progression, including immune evasion, angiogenesis promotion, and transfer of drug resistance (Zheng, Wang & Ge, 2025). In this study, we systematically investigated the transcriptomic alterations in HUVECs under dual stimulation by BCR-ABL overexpression and K562-derived exosomes, aiming to identify novel molecular signatures and potential therapeutic targets involved in microenvironmental remodeling in CML.

We employed transcriptome sequencing to analyze the differentially expressed genes (DEGs) in four groups: control HUVECs (Z), HUVECs overexpressing BCR/ABL (ZBA), HUVECs treated with K562-derived exosomes (Zexo), and HUVECs with both BCR-ABL overexpression and exosome treatment (ZBAexo). We identified seven specificized expressed genes in ZBAexo group: *CAMK2B*, *CDC25C*, *SV2A*, *MND1*, *CDC20*, *CLSPN*, and *GRM1*. Functional enrichment revealed that these genes are primarily involved in cell cycle regulation, DNA replication, and cell adhesion pathways—processes central to endothelial activation and microenvironment remodeling. Notably, their expression patterns were shaped by the combined stimulus of BCR-ABL signaling and leukemic exosomes, suggesting synergistic reprogramming of endothelial function. Network analysis indicated that transcription factors *CREB1* and *NFKB1* may regulate these seven genes, highlighting potential master regulators in the leukemic niche.

The calcium/calmodulin-dependent protein kinase II beta (*CAMK2B*), was upregulated in the ZBAexo group and enriched in pathways such as ECM-receptor interaction, complement and coagulation cascades, and cell adhesion, suggesting a role in enhancing endothelial-stromal interactions. The transcription factor *CREB1* is identified to have potential regulatory effects on *CAMK2B*, suggesting a complex regulatory network involving this gene in the context of CML. The expression of *CAMK2B* varies across different cancer types, CAMK2B has been reported as downregulated in gliomas and may act as a tumor suppressor in some contexts (Johansson, Göransson & Westermark, 2005). Its upregulation in this study may supports leukemic cell retention.

Cell Division Cycle 25C (*CDC25C*), is a phosphatase that activates cyclin-dependent kinases to drive cell cycle progression. Its overexpression is associated with poor prognosis in leukemia patients and inhibition of *CDC25C* can lead to cell cycle arrest and apoptosis. A novel mikanolide derivative has been found to exhibit potent antileukemic activity by inhibiting *CDC25C* phosphorylation, triggering apoptosis, and promoting DNA damage in leukemia cells (Rao et al., 2022). In this study, *CDC25C* was significantly downregulated in ZBAexo group, suggesting that leukemic signals may suppress cell cycle genes in endothelial cells. The regulatory interplay with miR-16-5p and lncRNAs AC008124.1 and AC064799.2 suggests a complex post-transcriptional network that may stabilize this altered state.

Synaptic vesicle glycoprotein 2A (SV2A) is a member of the major facilitator superfamily (MFS) of transport proteins and plays a crucial role in vesicle trafficking and exocytosis. In the ZBAexo group, SV2A was markedly downregulated, indicating that leukemic exosomes and BCR-ABL signaling may impair vesicular transport in endothelial cells. In triple-negative breast cancer (TNBC), *SV2A* has been identified as a biomarker that predicts differential responses to chemotherapy in patient-derived xenografts (Petrosyan et al., 2023). The regulatory landscape of *SV2A* is intricate, involving the transcription factor *CREB1* and the microRNA has-miR-16-5p, suggesting a critical checkpoint in *SV2A* expression control within CML cells. The inclusion of lncRNAs AC008124.1 and AC064799.2 in the *SV2A* regulatory network adds a further dimension, implying a complex ceRNA network. Emerging evidence indicates that exosomes derived from CML cells carry BCR-ABL mRNA and proteins, and can transfer drug resistance to sensitive cells (Zheng, Wang & Ge, 2025). The downregulation of SV2A—a vesicle trafficking protein—may reflect a broader reprogramming of exosome sorting in endothelial cells, potentially altering the composition of stromal-derived exosomes that feed back to leukemic cells.

Meiotic Nuclear Divisions 1 (*MND1*) plays a role in homologous recombination (HR) during meiosis and has been implicated in DNA repair in cancer. In this study, MND1 was downregulated in the ZBAexo group and enriched in DNA replication and cell cycle pathways. *MND1* is often overexpressed in various cancer types, including gastric cancer, and its high expression is linked to poor survival rates and advanced tumor stage (Koob et al., 2023). Recent studies have highlighted its role in tumorigenesis, particularly in leukemia. In leukemia, *MND1* interacted with other proteins such as TKT to regulate tumor progression, and it modulated chemotherapy sensitivity by PI3K/AKT signaling pathway (Hu et al., 2023). Notably, BCR-ABL+ cells exhibit increased genomic instability and altered DNA repair mechanisms, the suppression of MND1 in endothelial cells could be a consequence of this systemic DNA repair dysregulation, further promoting a pro-tumorigenic niche.

Cell Division Cycle 20 (*CDC20*), an activator of the anaphase-promoting complex/cyclosome (APC/C), promots the degradation of proteins that are essential for the maintenance of the mitotic checkpoint and the progression of cell division. *CDC20* was downregulated in the ZBAexo group, high levels of *CDC20* have been observed in various leukemia subtypes and are associated with poor prognosis (Manoochehrabadi et al., 2024). It is a ubiquitination-related gene associated with immune cell infiltration in CML, and its high expression in leukemia stem cells (CML-LSCs) contributes to cell proliferation. Knockdown of *CDC20* leads to significant inhibition of CML cell growth (Zhou et al., 2024). *CDC20* regulate the progression of CML by regulating cell cycle proteins and influencing the response to tyrosine kinase inhibitors (Zhou et al., 2024). In this study, the transcription factor *NFKB1* is implicated in the regulation of *CDC20*. NFKB1 is a key mediator of inflammation, suggests that CDC20 may be part of a broader inflammatory network that sustains the leukemic niche.

Claspin (*CLSPN*) is a multifunctional protein that plays a critical role in cell homeostasis, particularly in processes related to DNA replication, damage response, and cell cycle regulation (Yabushita et al., 2023). *CLSPN* has emerged as a key player in the mechanism of action of decitabine, a drug used to treat myelodysplastic syndrome (MDS) and acute myeloid leukemia (AML). In this study, CLSPN was downregulated in the ZBAexo group, indicating potential impairment in DNA repair mechanisms in endothelial cells. *CLSPN* mediate the response to decitabine through aberrant *DNMT1*-DNA covalent bonds (Yabushita et al., 2023). Beyond leukemia, *CLSPN* has been implicated in the progression of solid tumors. Moreover, *CLSPN* overexpression has been linked to cisplatin resistance in urothelial carcinoma, highlighting its potential as a target for immunotherapy (Yamada et al., 2023). These results may suggest that *CLSPN* appears to play a pivotal role in the development of resistance to chemotherapeutic agents.

The metabotropic glutamate receptor 1 (GRM1) is a G protein-coupled receptor involved in CNS signaling (Eddy & Chen, 2021). In melanoma, GRM1 has been identified as a significant driver of tumor growth and progression. Activation of GRM1 stimulates downstream signaling pathways, such as the MAPK and PI3K/AKT pathways. This activation leads to increased cell proliferation, survival, and metastasis. Its activation is associated with proangiogenic signaling and poor clinical outcomes, indicating its potential as a therapeutic target (Teh & Chen, 2012). The transcription factor *NFKB1* and the microRNA has-miR-126-5p, along with the gene *AGAP11*, form a regulatory network that may influence *GRM1* expression in CML, suggesting a complex interplay of regulatory elements in the disease’s molecular profile. Therefore, the multifaceted role of *GRM1* in cancer biology positions it as a promising target for the development of targeted therapies aimed at disrupting key oncogenic processes.

*CAMK2B* and *CDC25C* are prominently involved in cell cycle regulation, are regulated by the transcription factor *CREB1*. *CDC25C*, is targeted by miR-16-5p, which influences its expression and potential role in leukemia progression. *SV2A* and *MND1* participate in DNA replication. *SV2A* is also regulated by *CREB1* and miR-16-5p, indicating a multi-layered regulate mechanism. *MND1* interacts with TKT and influences the PI3K/AKT pathway, implicating it in DNA repair and chemosensitivity modulation. *CDC20* and *CLSPN* play roles in cell cycle progression and checkpoint control. *CDC20,* regulated by *NFKB1* and negatively correlated with BCR-ABL, is a hub for ubiquitin-mediated cell cycle control and stem cell proliferation*. CLSPN* interact with *DNMT1* to mediate decitabine response in leukemia. Lastly, *GRM1* is engaged in cell adhesion processes, and is regulated by *NFKB1*, miR-126-5p, and *AGAP11*, highlighting the complexity of its regulatory environment. Together, these genes form a transcriptionally and post-transcriptionally coordinated network shaped by exosomes and BCR-ABL, highlighting their potential as targets to disrupt microenvironment-mediated resistance in CML.

## Conclusions

In our comprehensive study, we have identified seven genes—*CAMK2B*, *CDC25C*, *SV2A*, *MND1*, *CDC20*, *CLSPN*, and *GRM1*—that exhibit significant correlations with the BCR-ABL fusion gene. They are integral to various key biological pathways that are essential in the development and progression of CML. Also, they are part of a sophisticated regulatory network that includes interacting transcription factors and microRNAs.

Despite these insights, several limitations must be acknowledged. First, our model uses HUVECs, which may not fully recapitulate the behavior of bone marrow endothelial cells. Second, the findings have not been validated in independent external datasets or clinical cohorts. The dysregulation of the seven genes remains to be confirmed at the protein level and in primary bone marrow endothelial cells from CML patients. Additionally, public databases such as GEO or TCGA currently lack comprehensive transcriptomic profiles of vascular niche cells in CML, limiting cross-validation. Therefore, while our integrative bioinformatics approach provides strong mechanistic hypotheses, future studies should aim to validate these results using patient-derived samples to strengthen their biological and clinical relevance.

##  Supplemental Information

10.7717/peerj.20371/supp-1 Supplemental Information 1 BCR-ABL Transformation of HUVEC cells and cell function analysis

10.7717/peerj.20371/supp-2 Supplemental Information 2 Exosome analysis and validation (A) Nanoparticle tracking analysis (NTA) for exosome size) characterization; (B) Western blot detection of exosome-specific marker proteins.

10.7717/peerj.20371/supp-3 Supplemental Information 3 Principal component analysis of four groups of samples

10.7717/peerj.20371/supp-4 Supplemental Information 4 Differential gene analysis of four groups of samples

10.7717/peerj.20371/supp-5 Supplemental Information 5 Analysis of genes related to cell adhesion, angiogenesis, and apoptosis

10.7717/peerj.20371/supp-6 Supplemental Information 6 Analysis of differentially expressed miRNAs and lncRNAs

10.7717/peerj.20371/supp-7 Supplemental Information 7 MIQE checklist

10.7717/peerj.20371/supp-8 Supplemental Information 8 Prediction of small molecule targeted drugs for the genes CAMK2B, CDC25C, SV2A, and GRM1

10.7717/peerj.20371/supp-9 Supplemental Information 9 Method of qRT-PCR

10.7717/peerj.20371/supp-10 Supplemental Information 10 Gene expression data of mRNA, miRNA, cirRNA and lncRNA

## Additional Information and Declarations

### Data Availability

The following information was supplied regarding data availability: The gene expression data are available in the Supplementary Materials. The raw RNA-seq data are available at the Sequence Read Archive (SRA) database: SRP553619.
