# Supplementary figures and images for: Exosome and BCR-ABL mediated molecular alterations in endothelial cells in chronic myeloid leukemia: identification of seven genes and their regulatory network
Source: PeerJ. 2025 Dec 17;13:e20371. doi: 10.7717/peerj.20371 (PMC12717845; doi:10.7717/peerj.20371)

**A**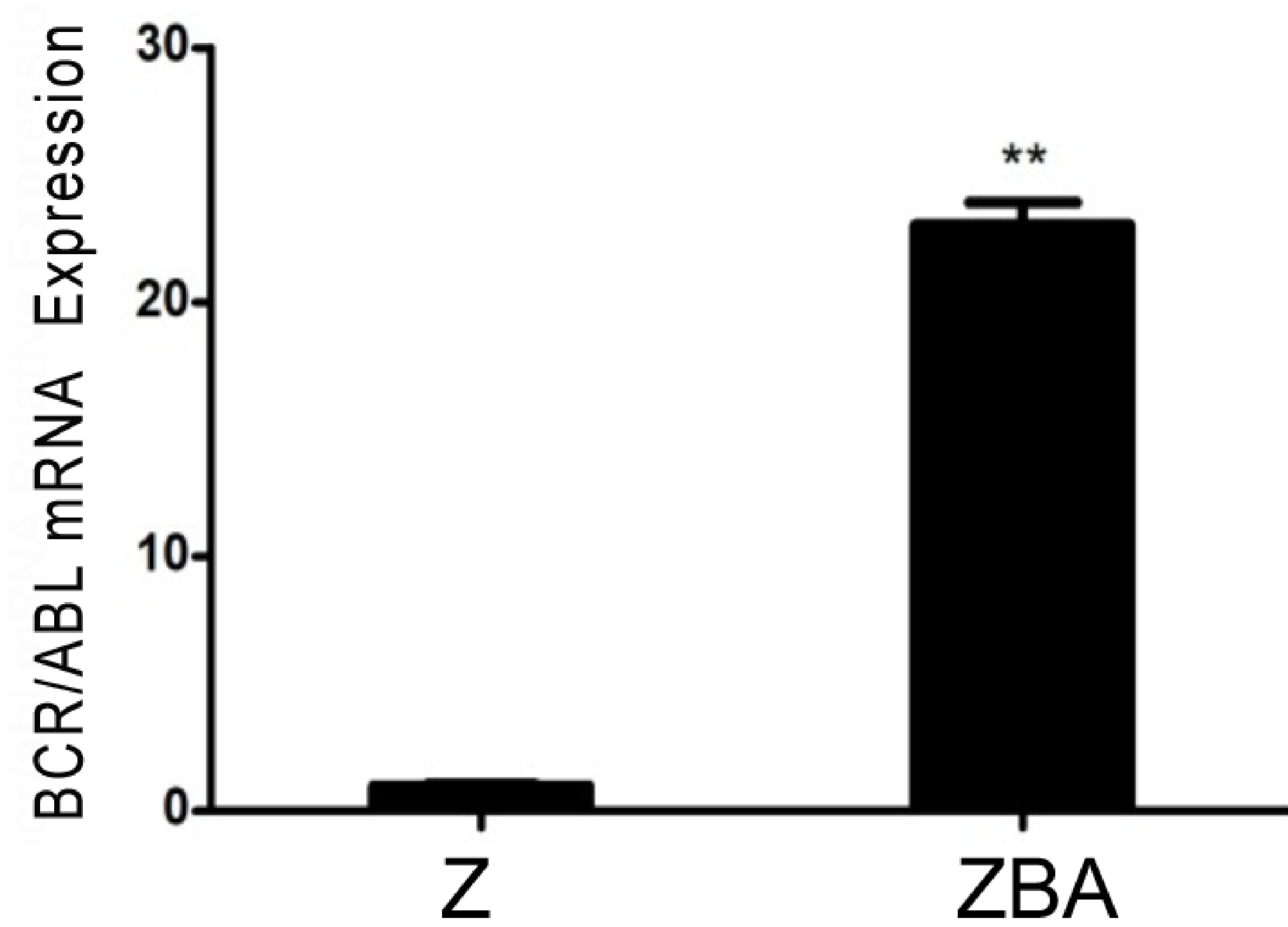**B**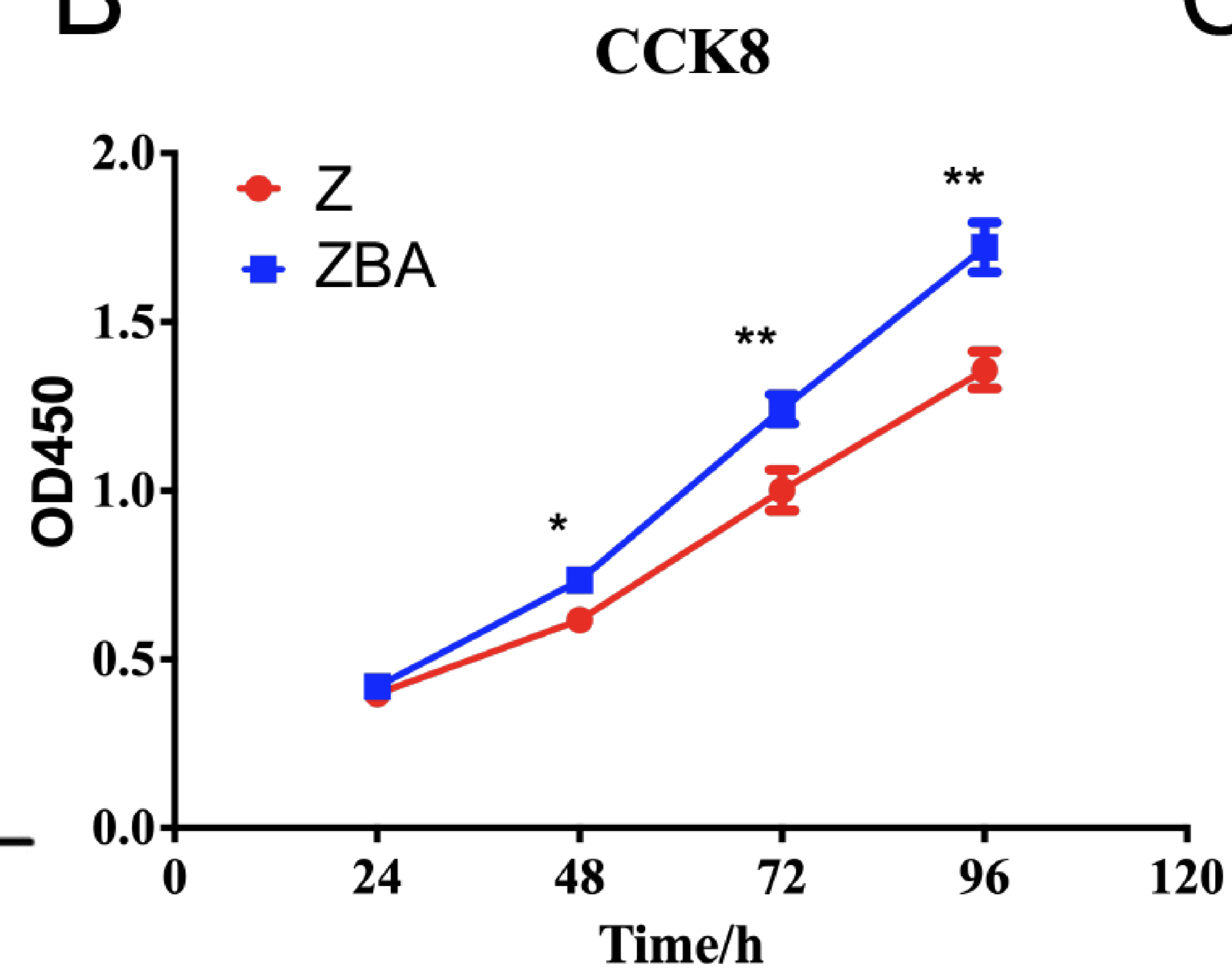**C**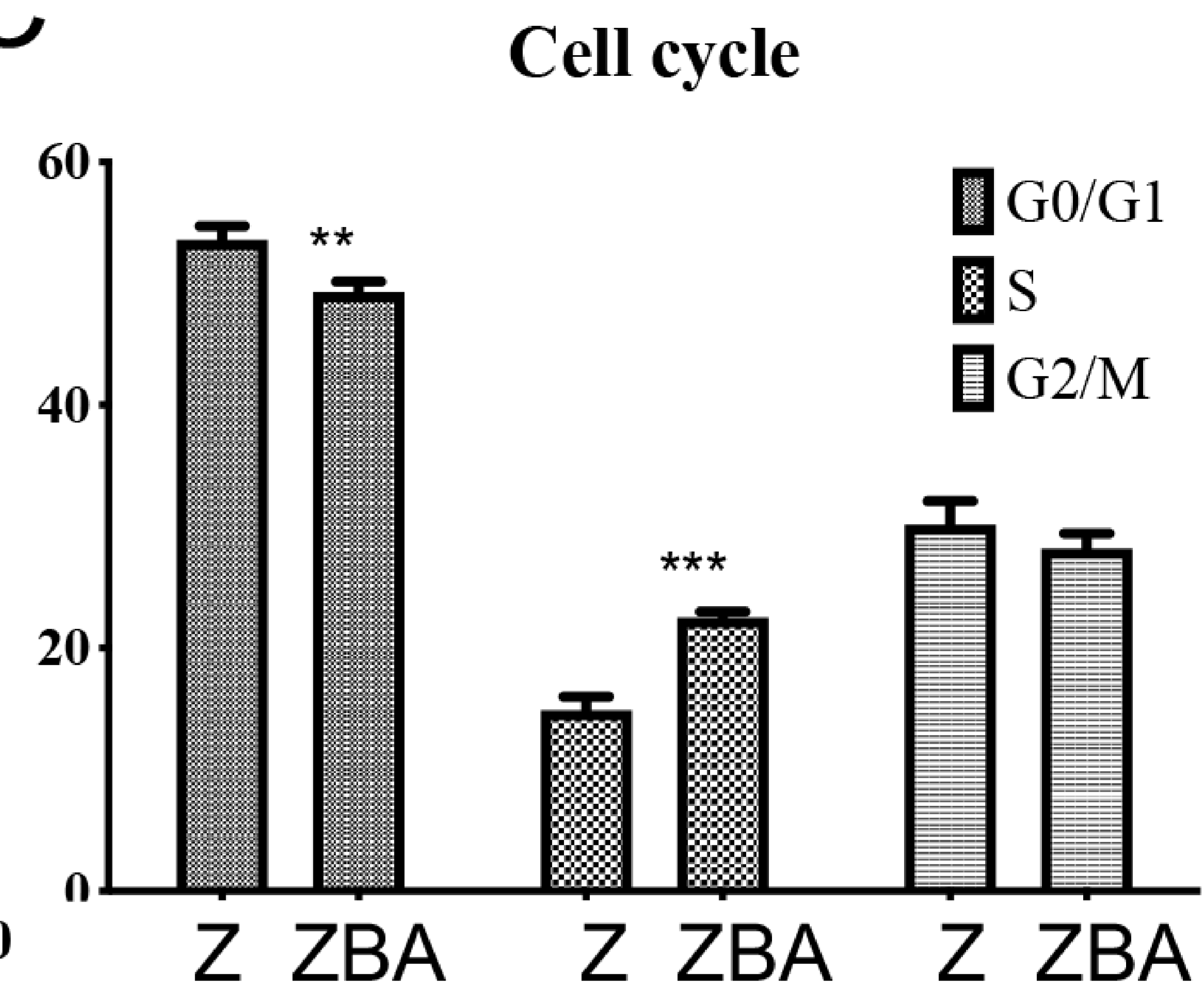**D**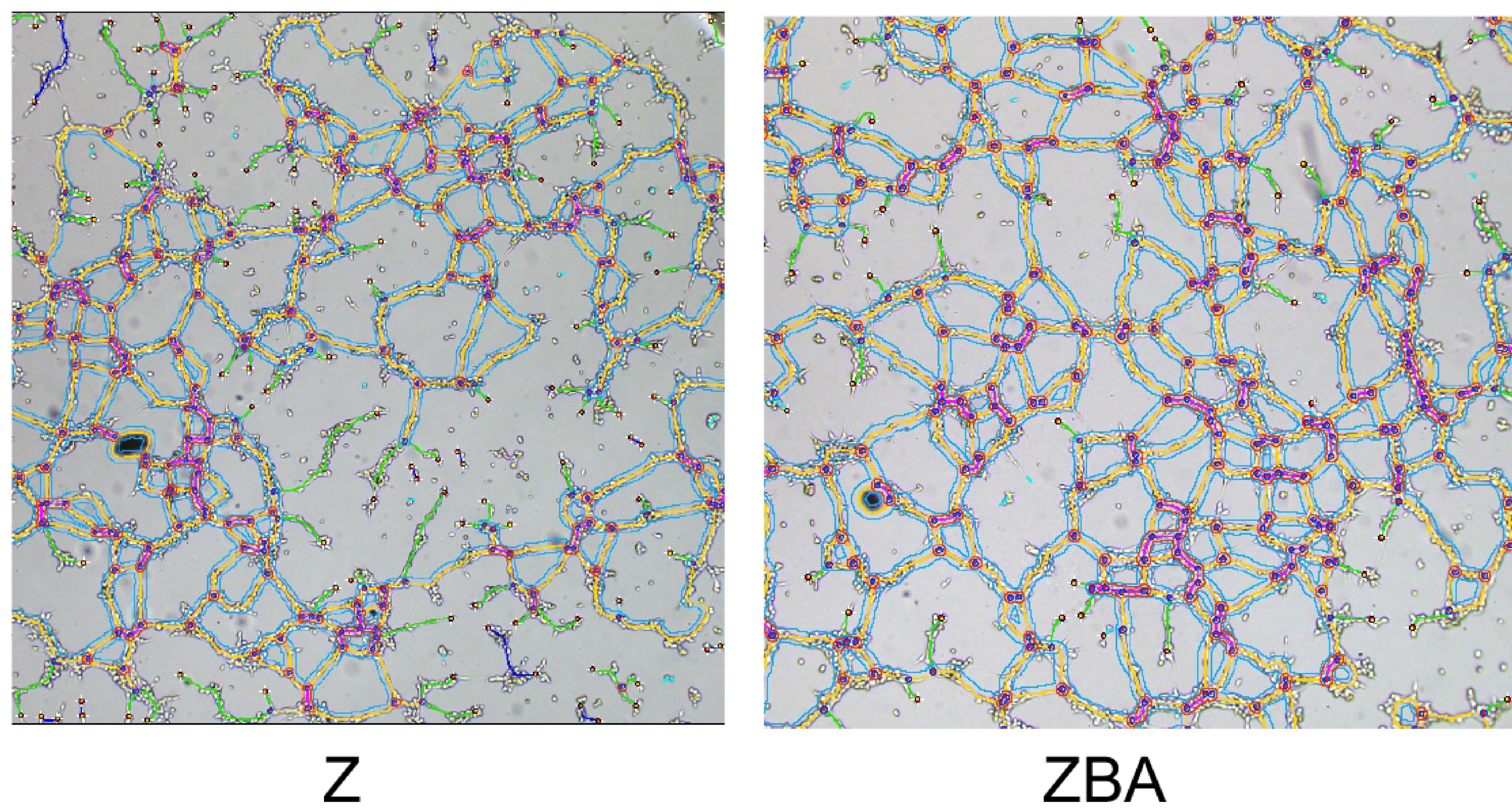**E**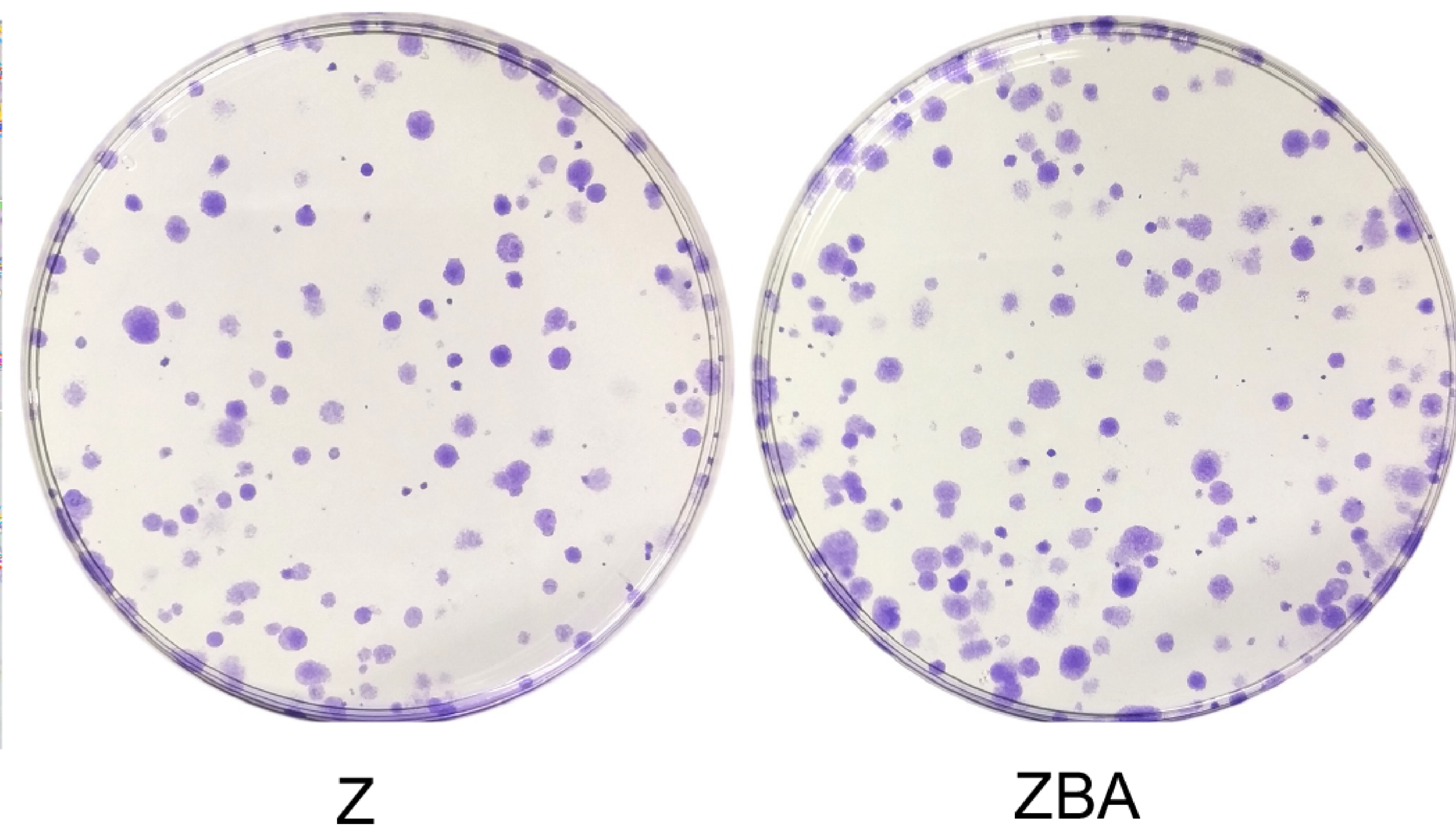**F**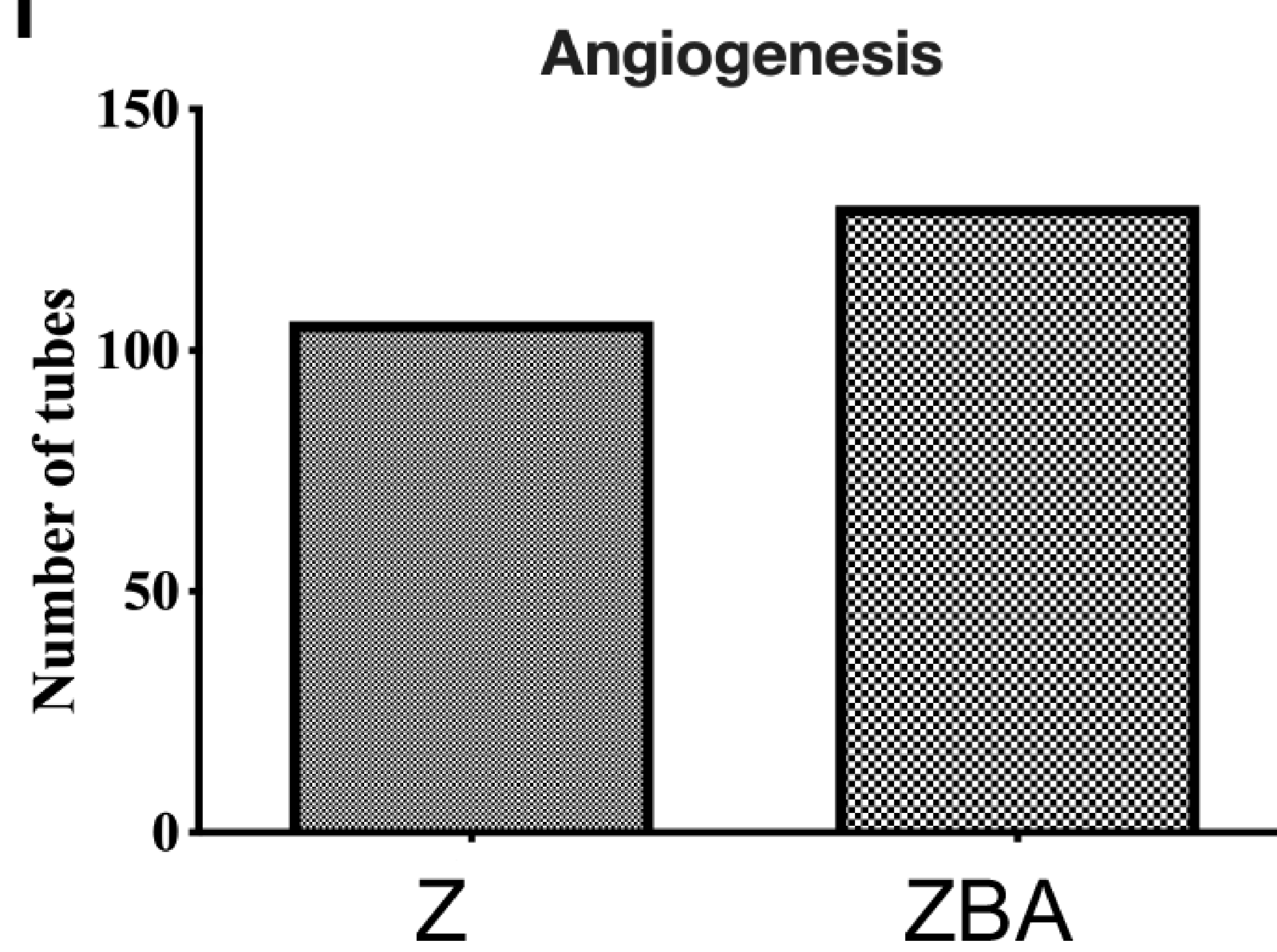**G**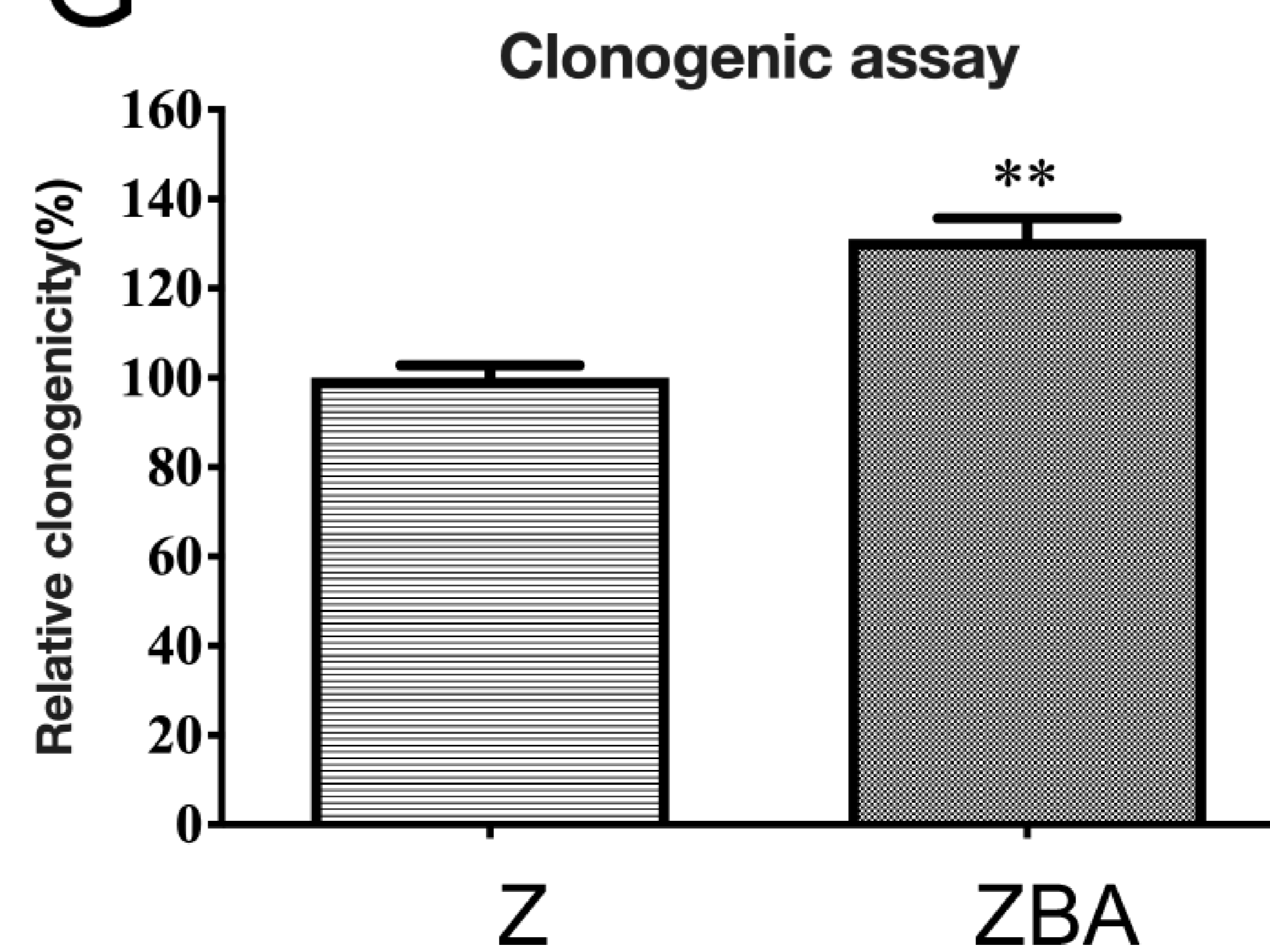**H**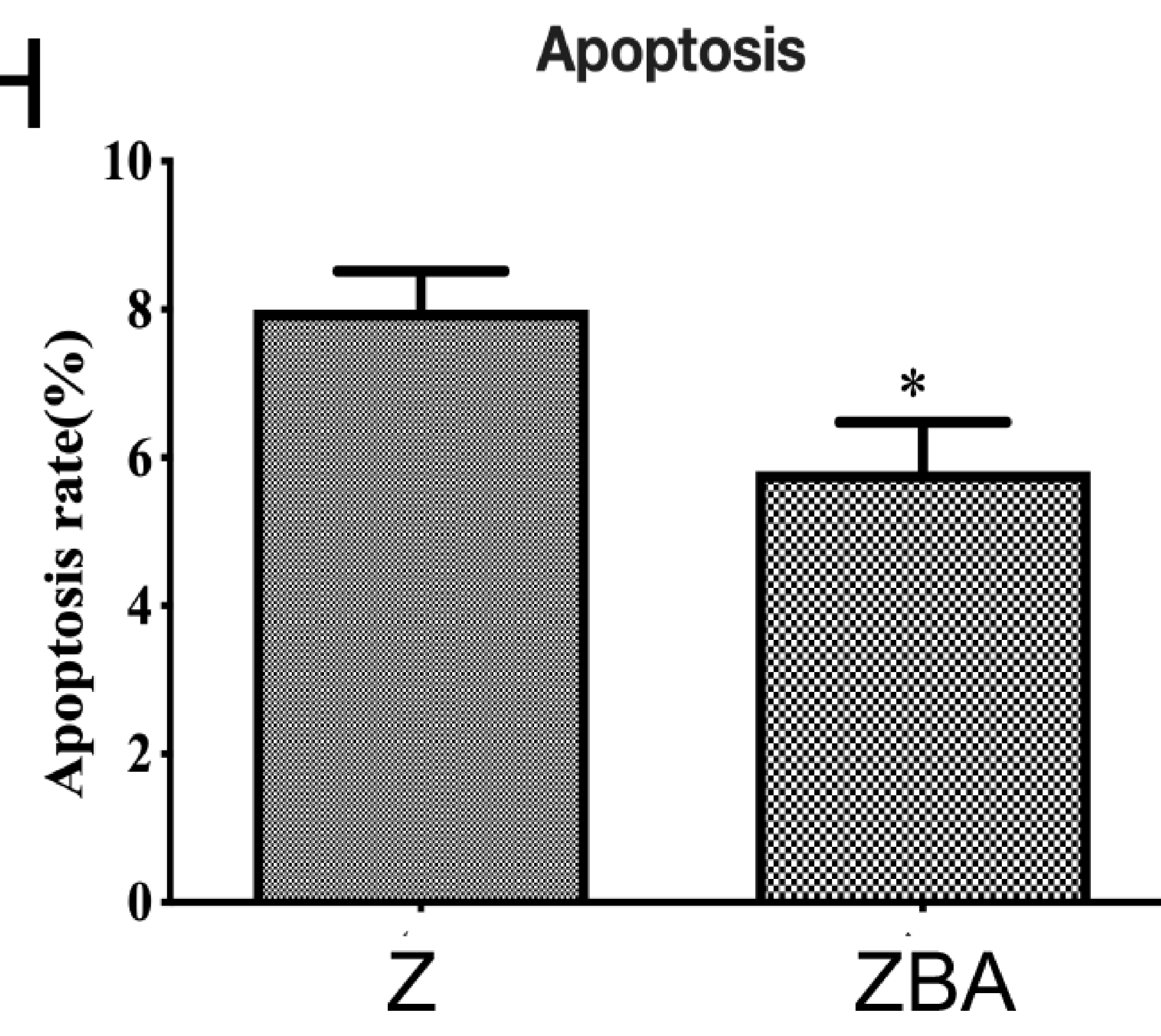

Supplement: Supplemental Information 1 [file peerj-13-20371-s001.pdf]

**A**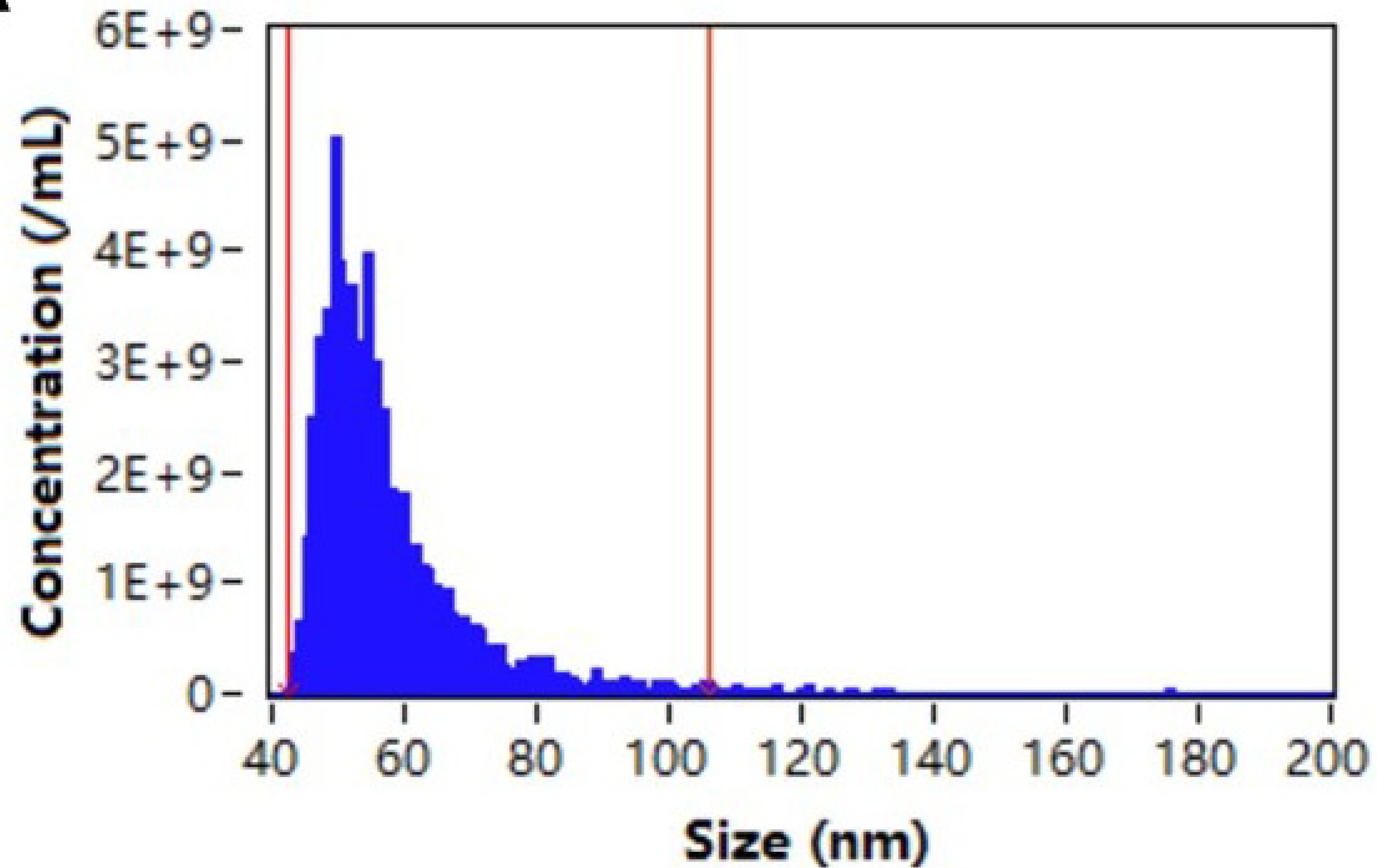**B**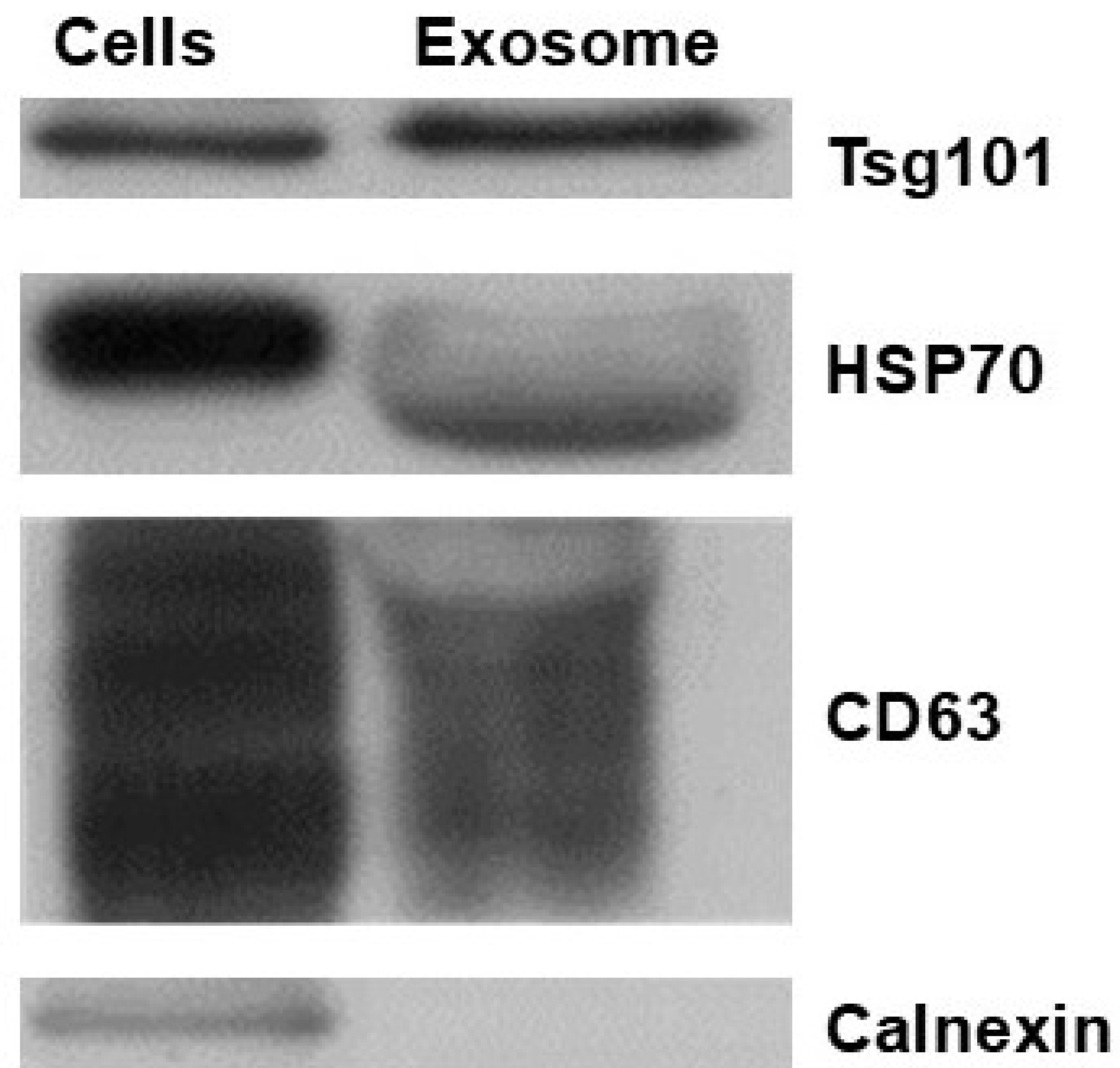

Supplement: Supplemental Information 2 — (A) Nanoparticle tracking analysis (NTA) for exosome size) characterization; (B) Western blot detection of exosome-specific marker proteins. [file peerj-13-20371-s002.pdf]

# Individuals – PCA

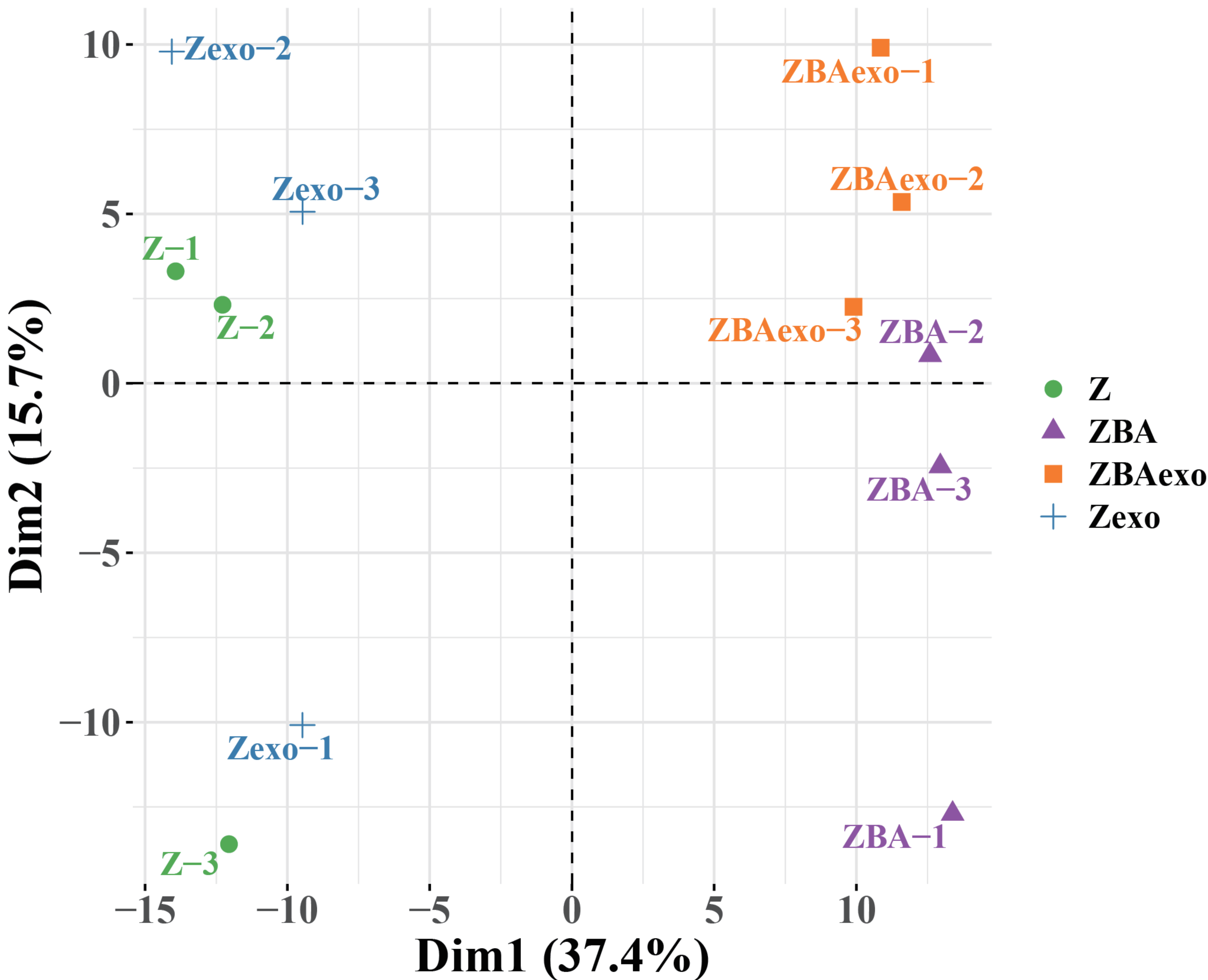

Supplement: Supplemental Information 3 [file peerj-13-20371-s003.pdf]

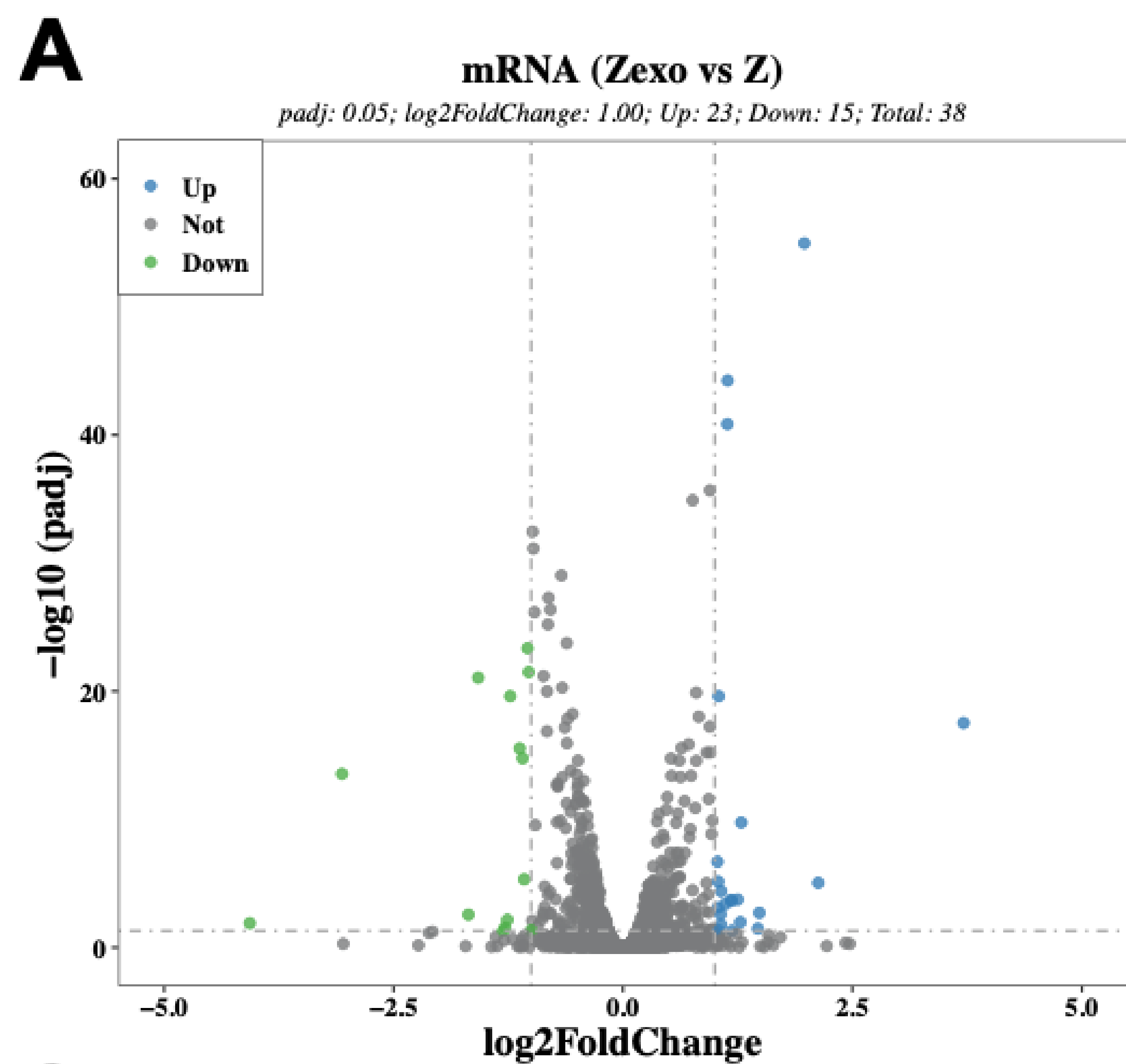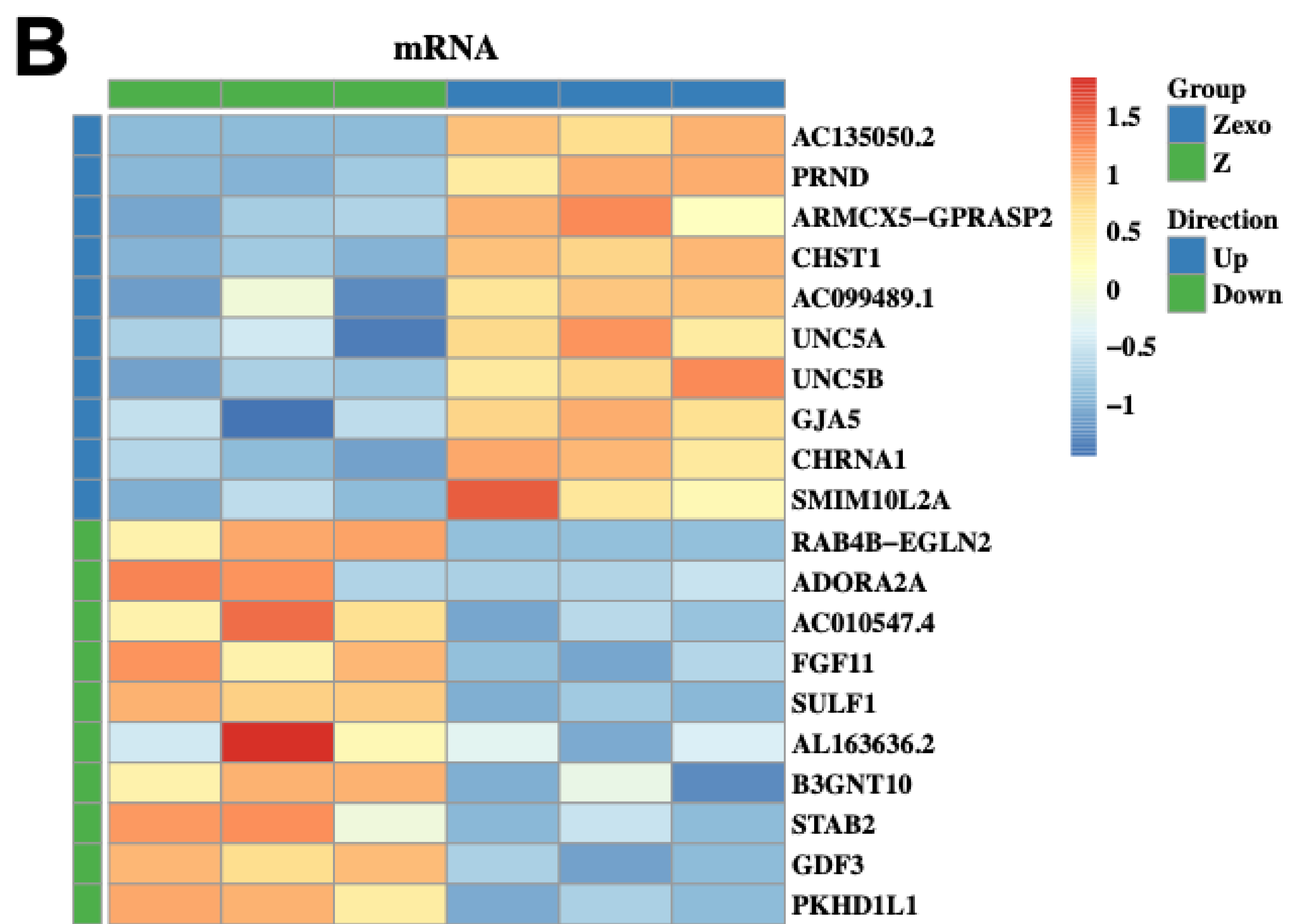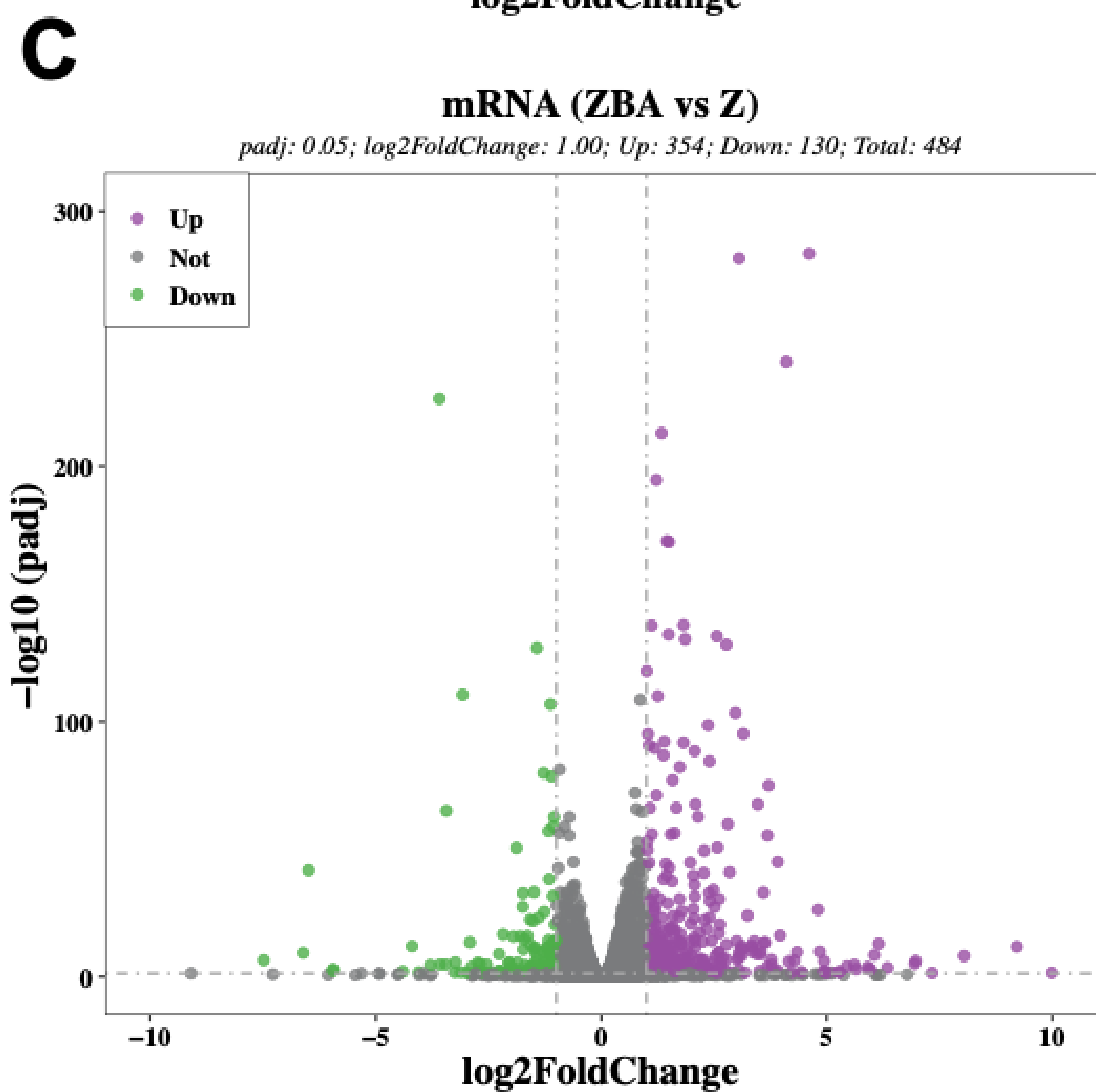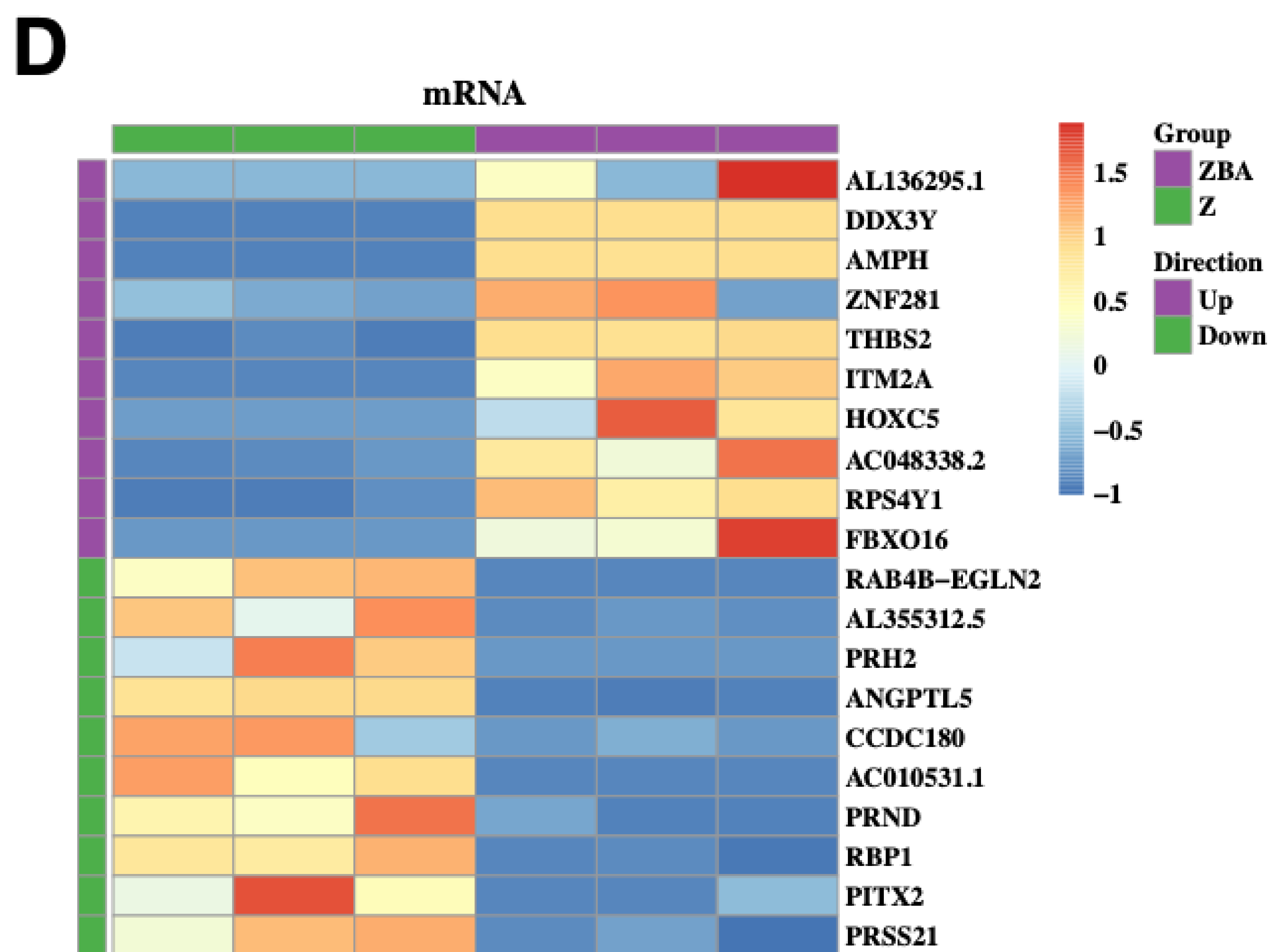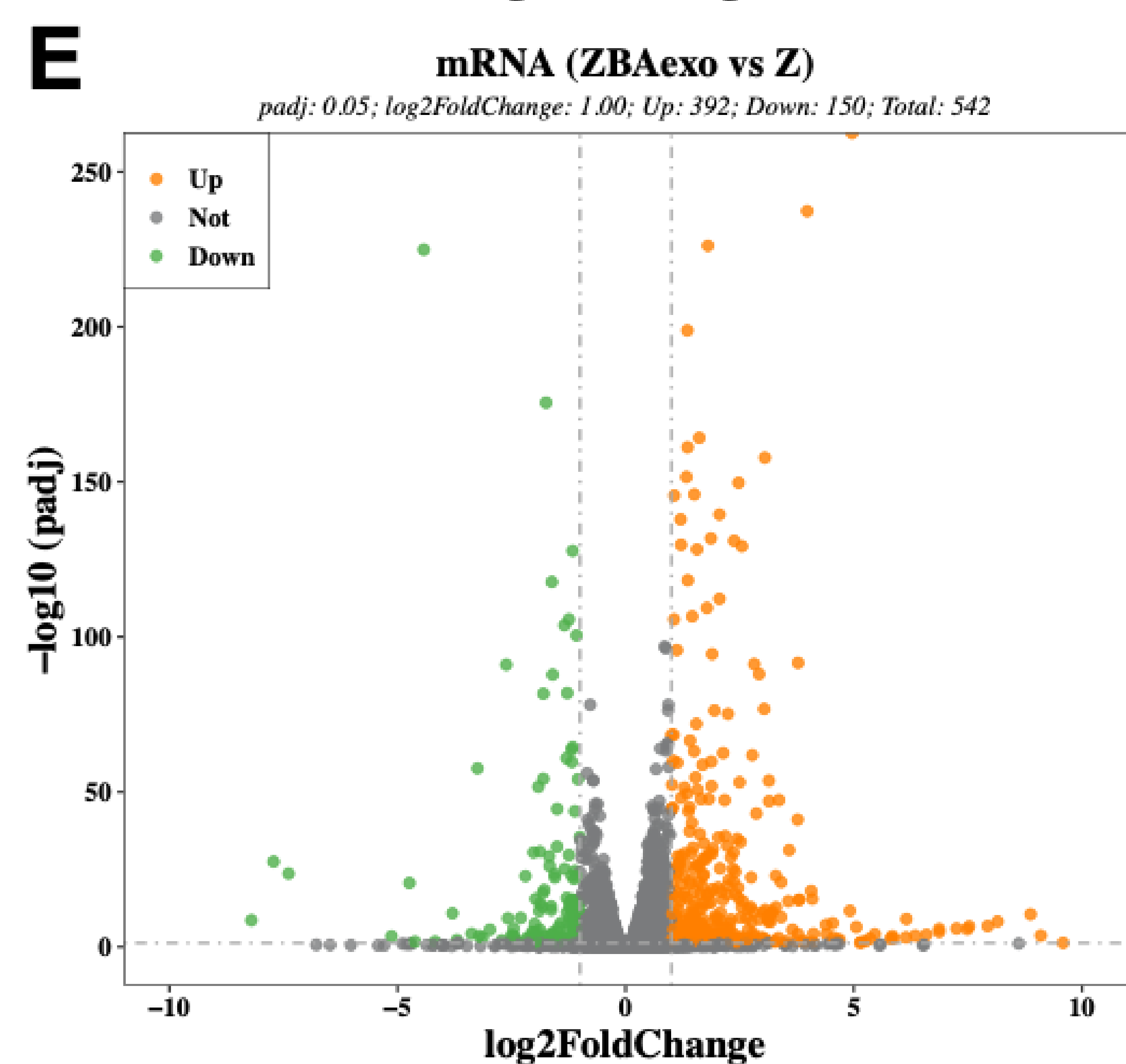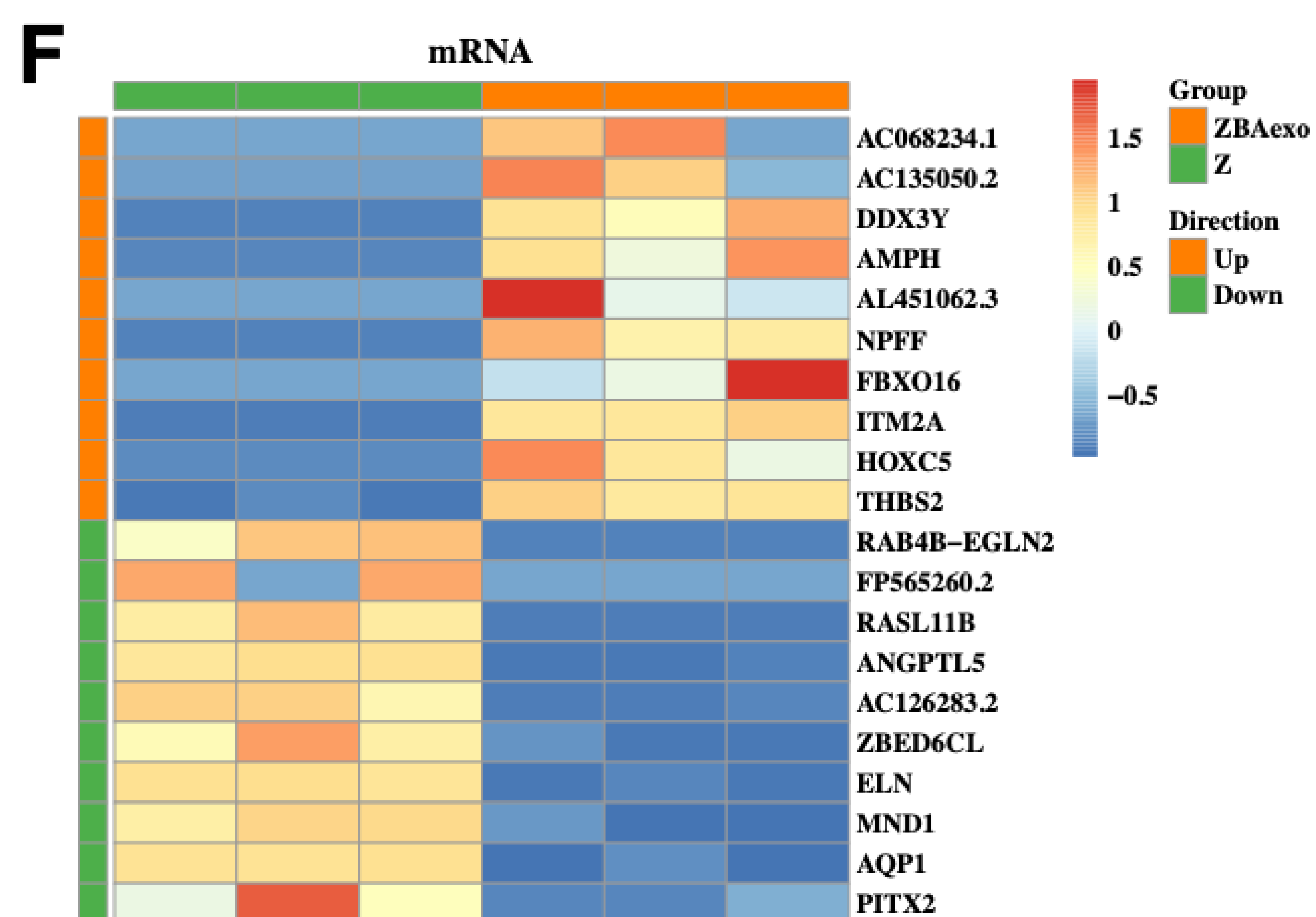

Supplement: Supplemental Information 4 [file peerj-13-20371-s004.pdf]

A

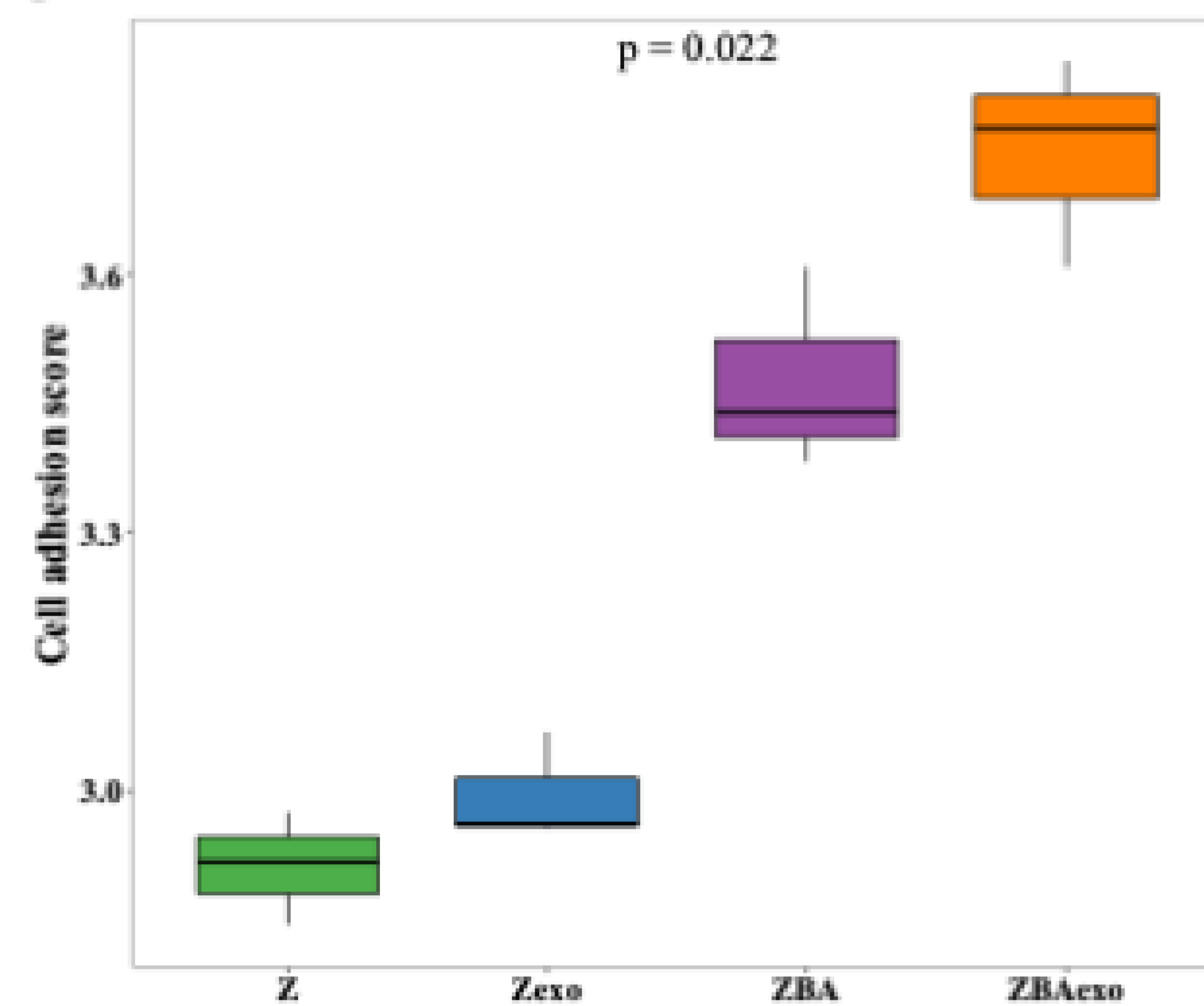

B

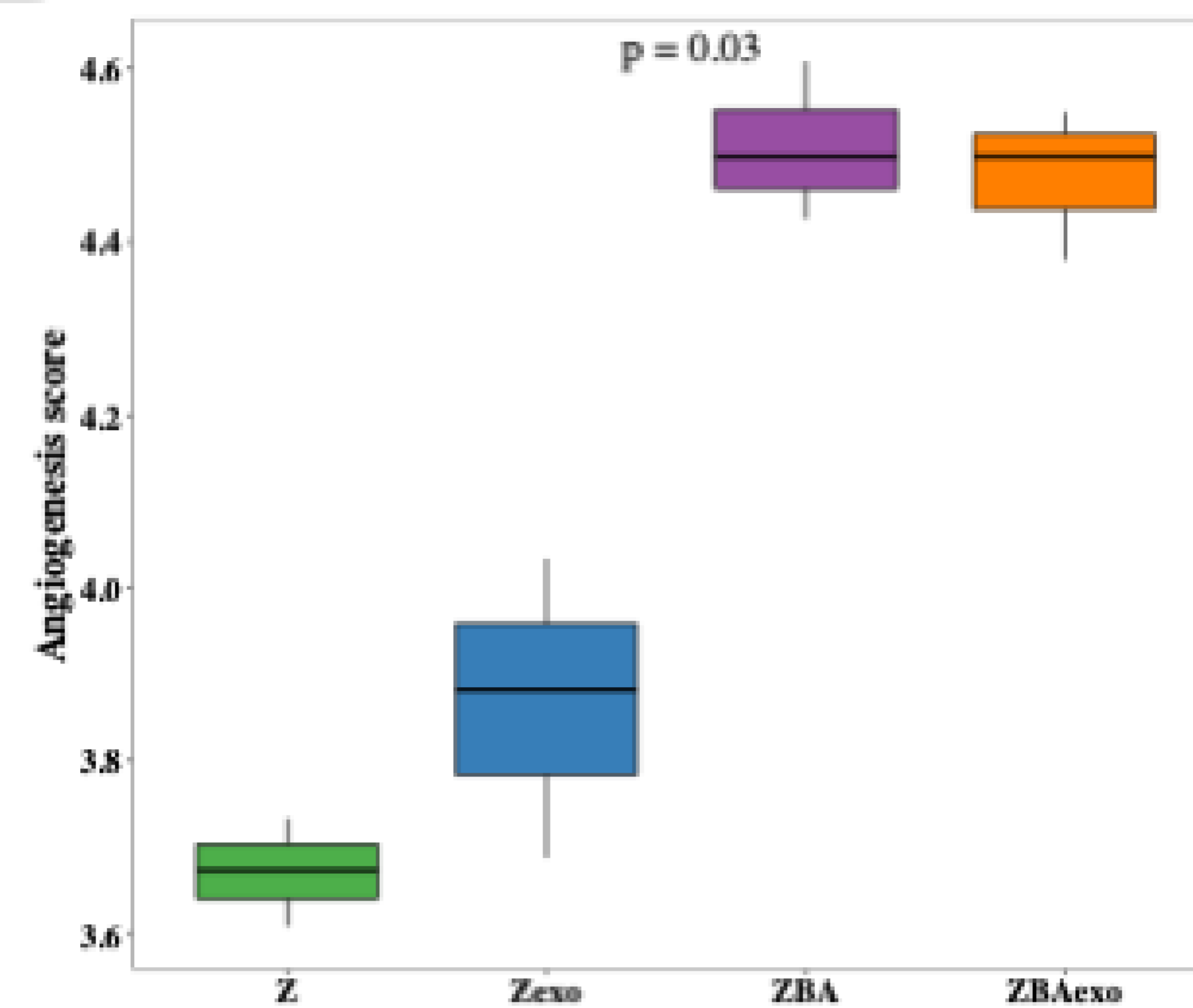

C

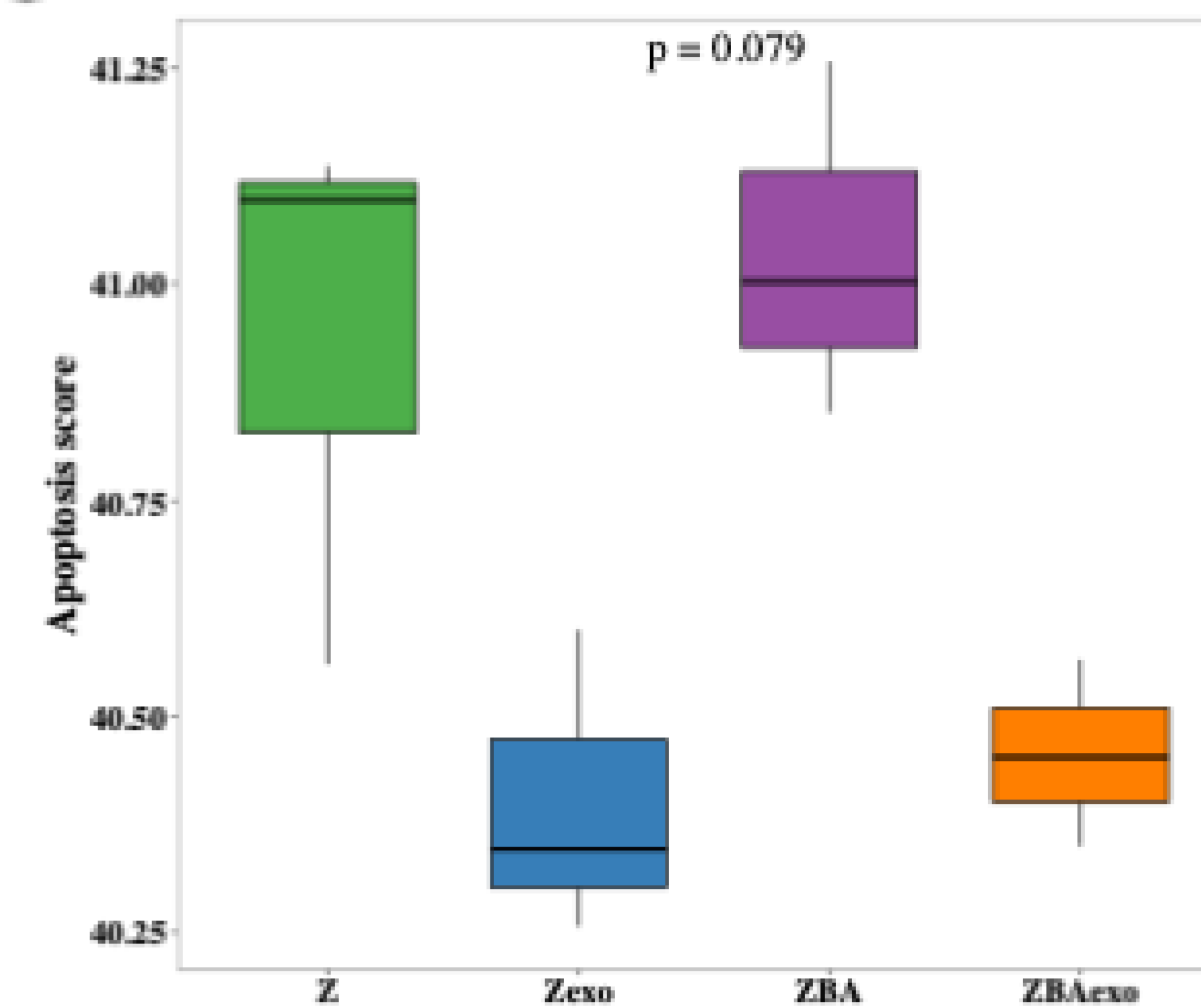

D

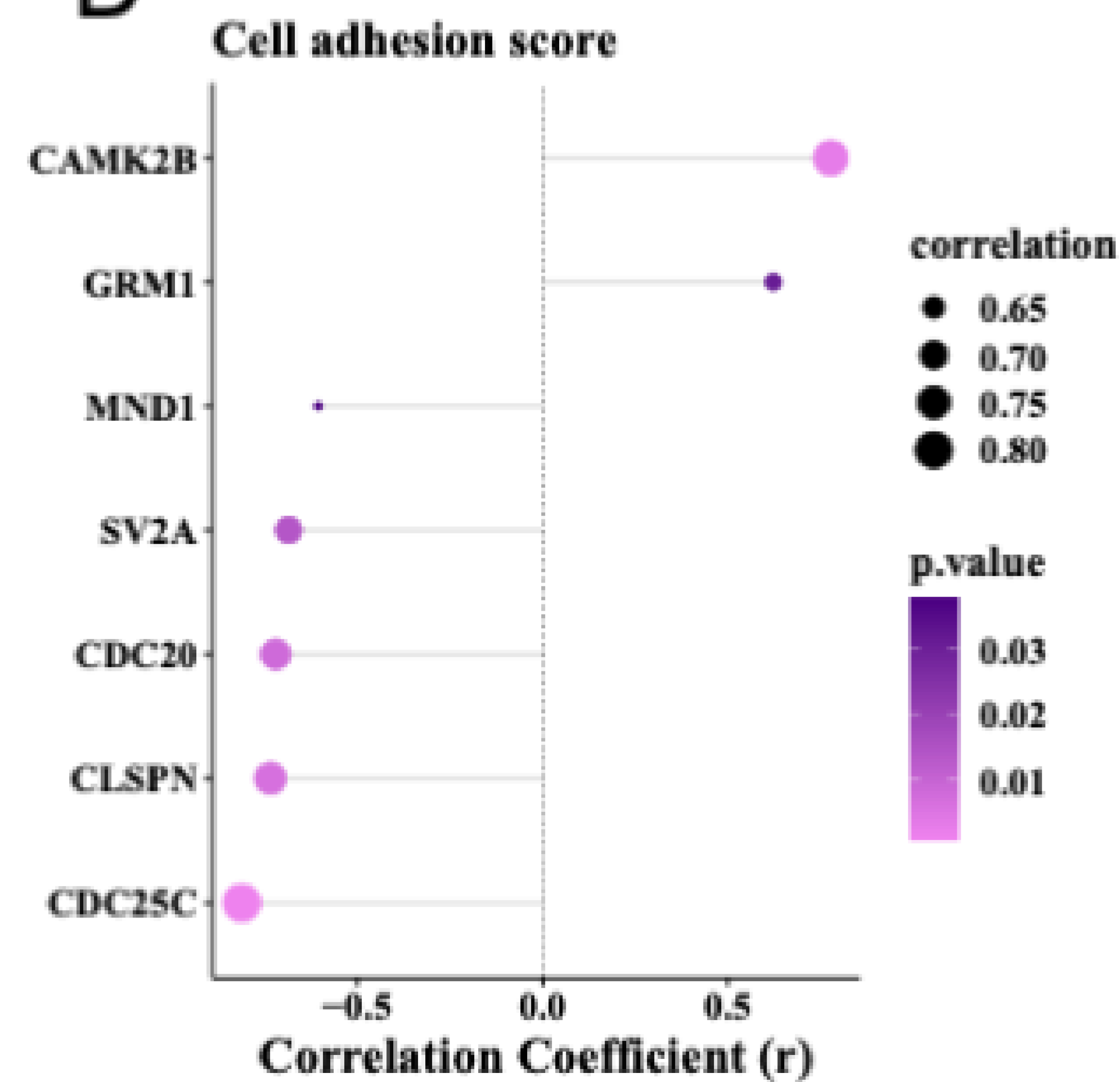

E

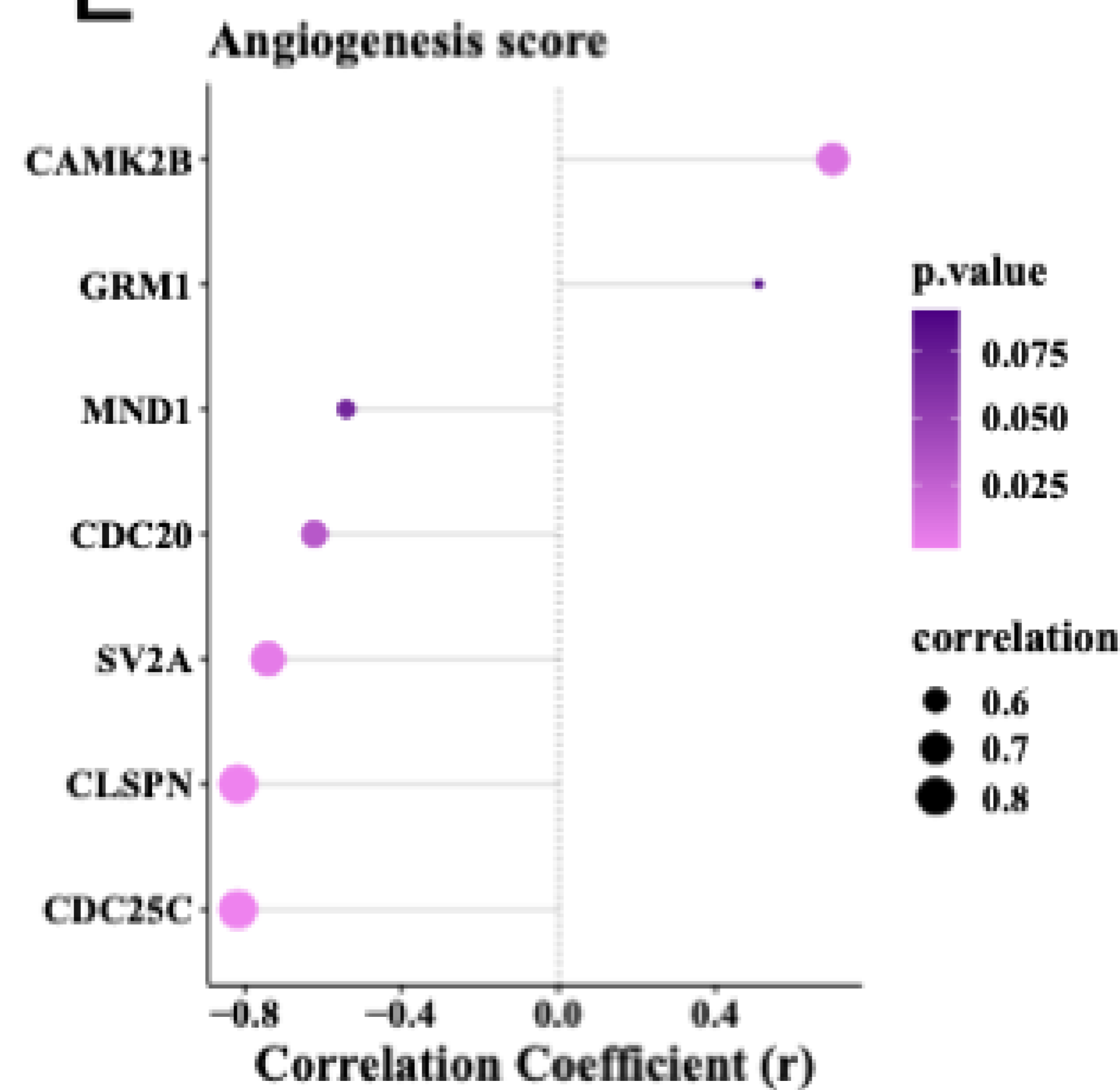

F

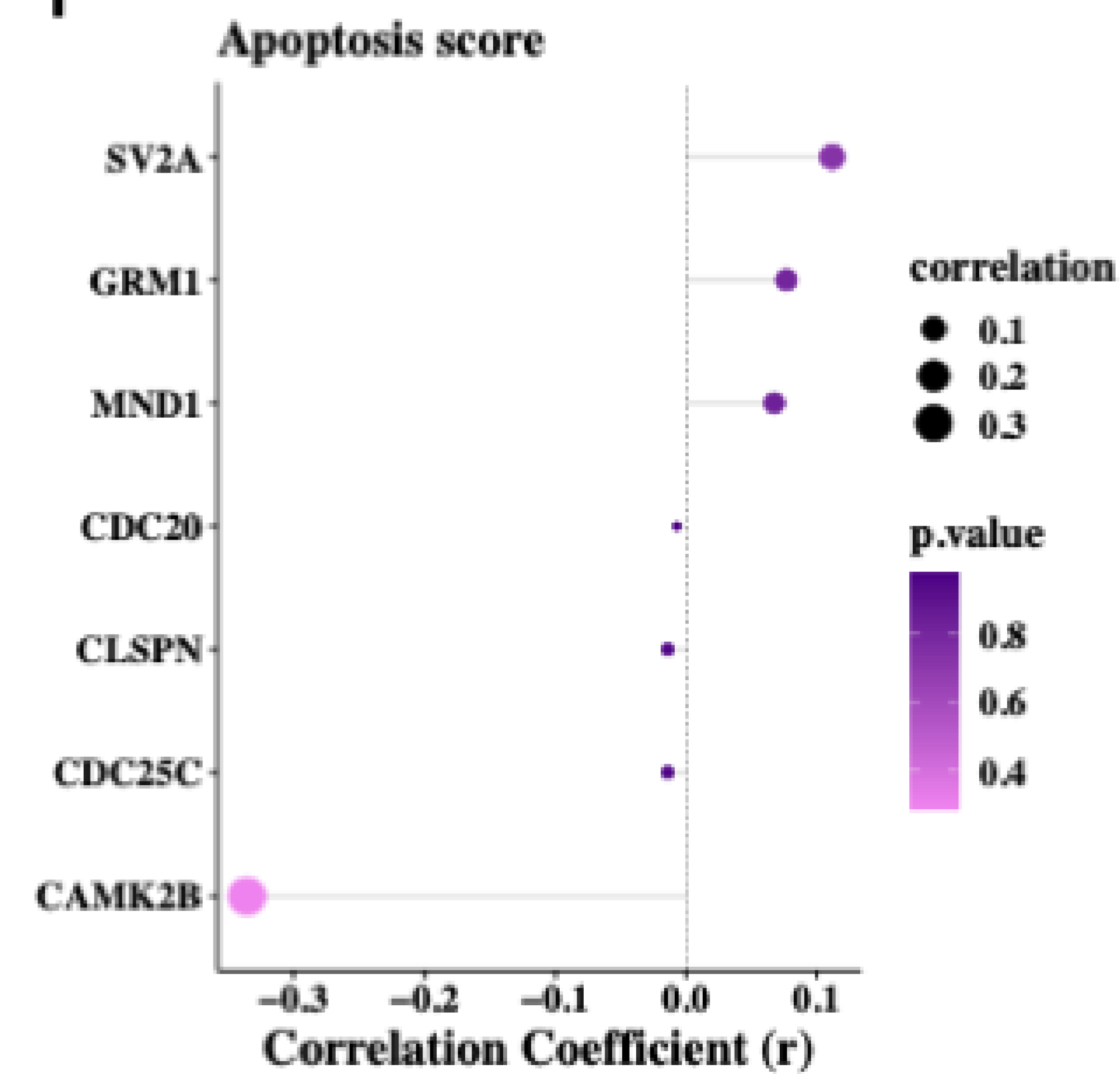

Supplement: Supplemental Information 5 [file peerj-13-20371-s005.pdf]

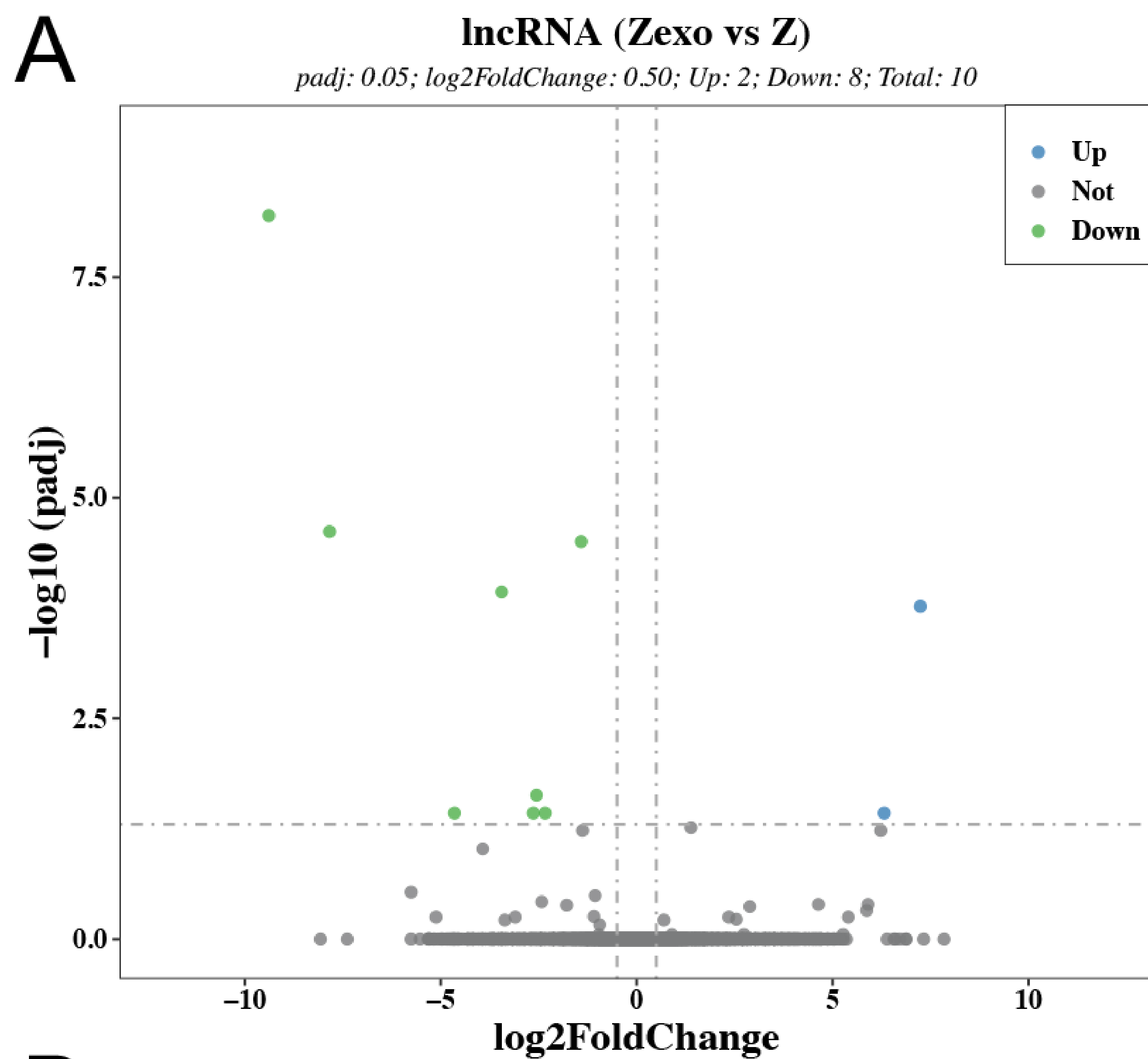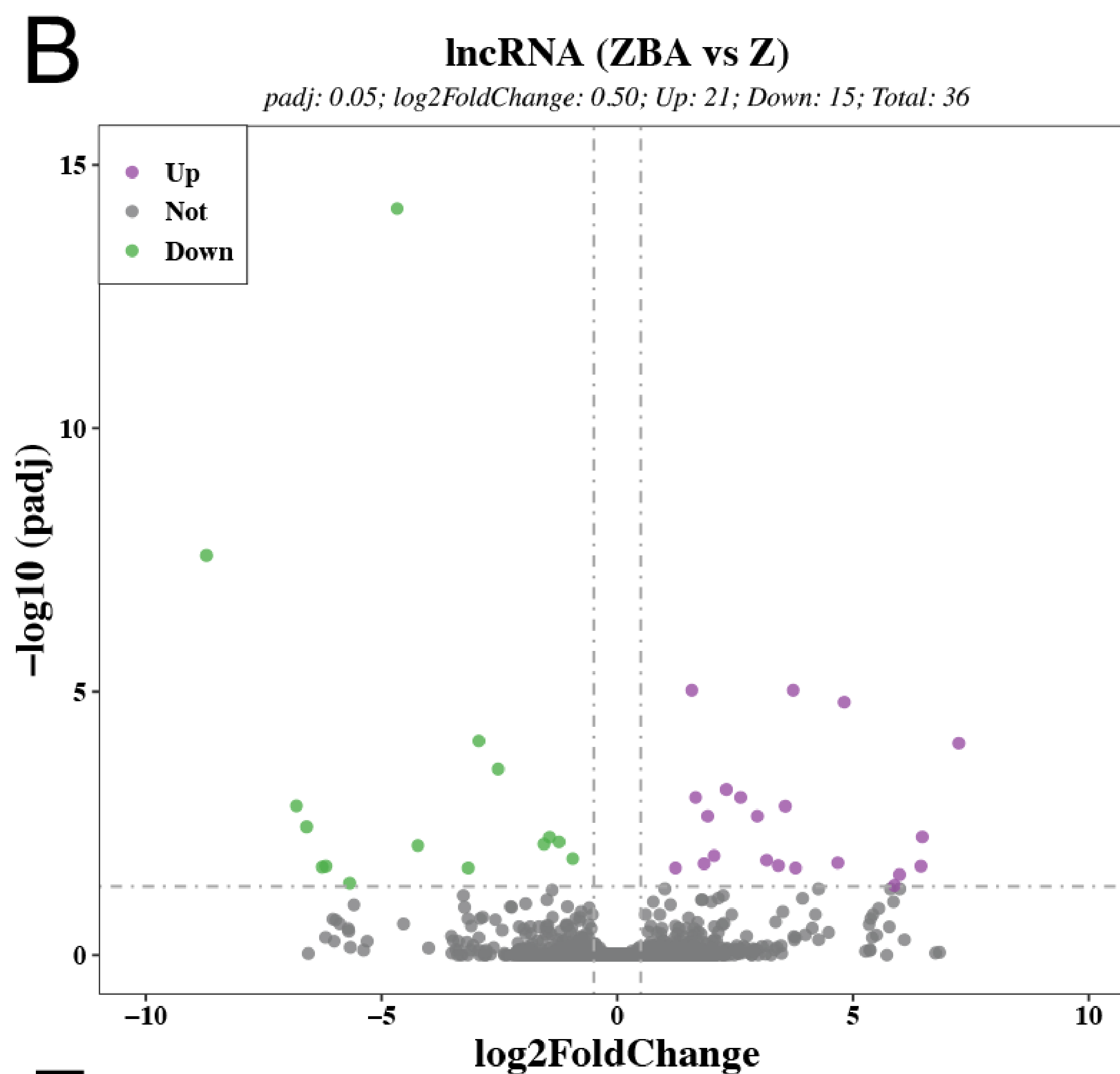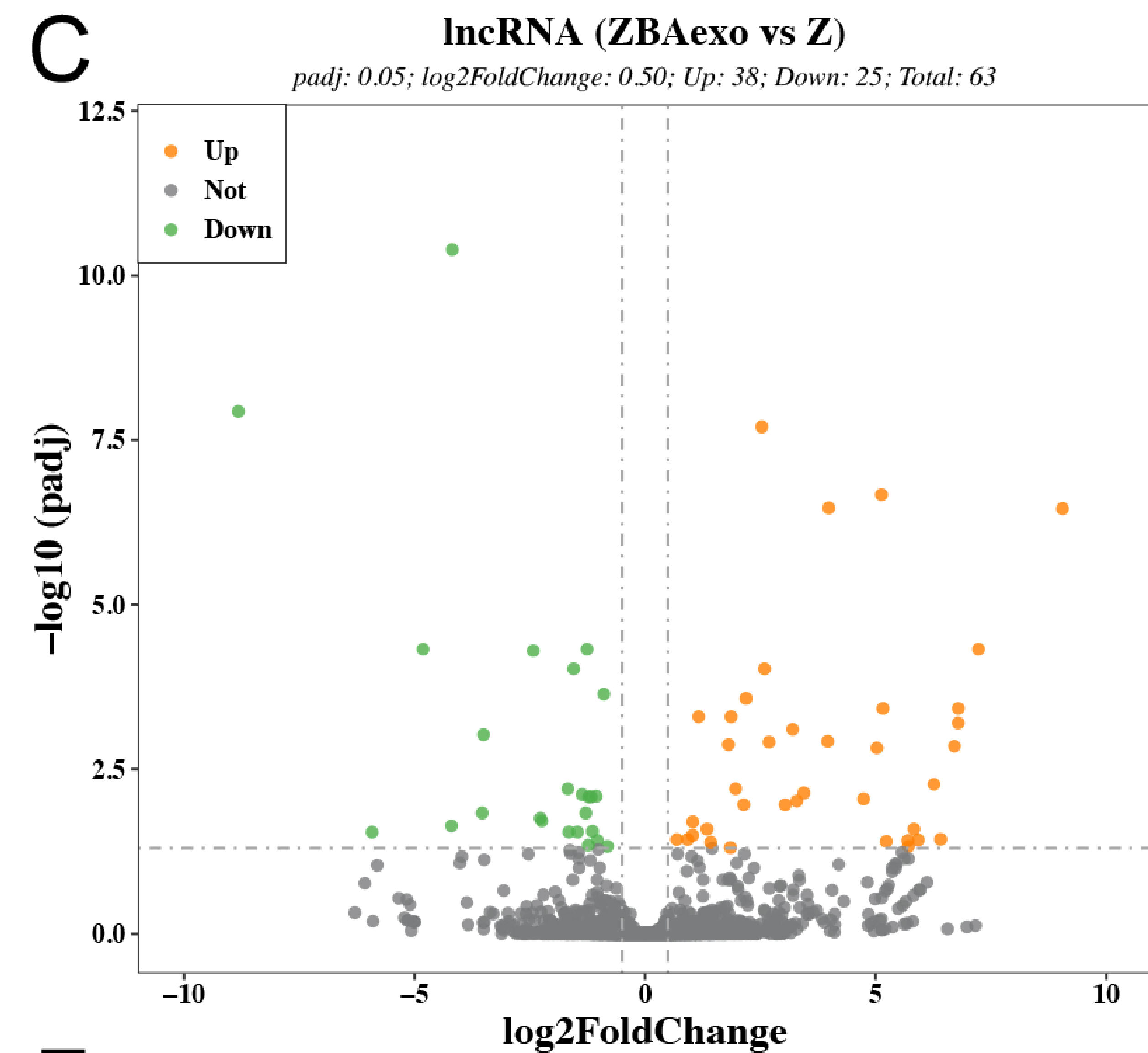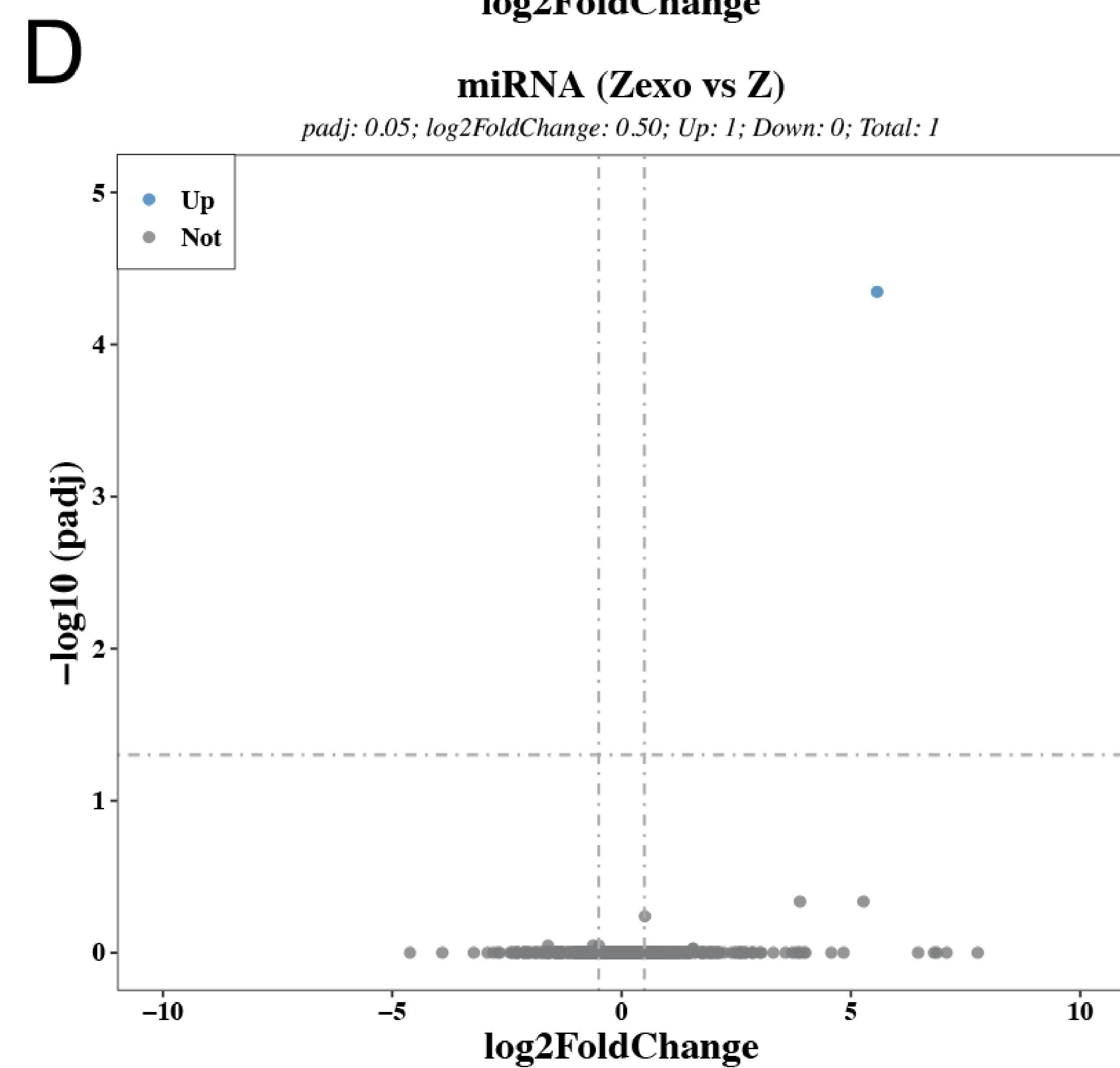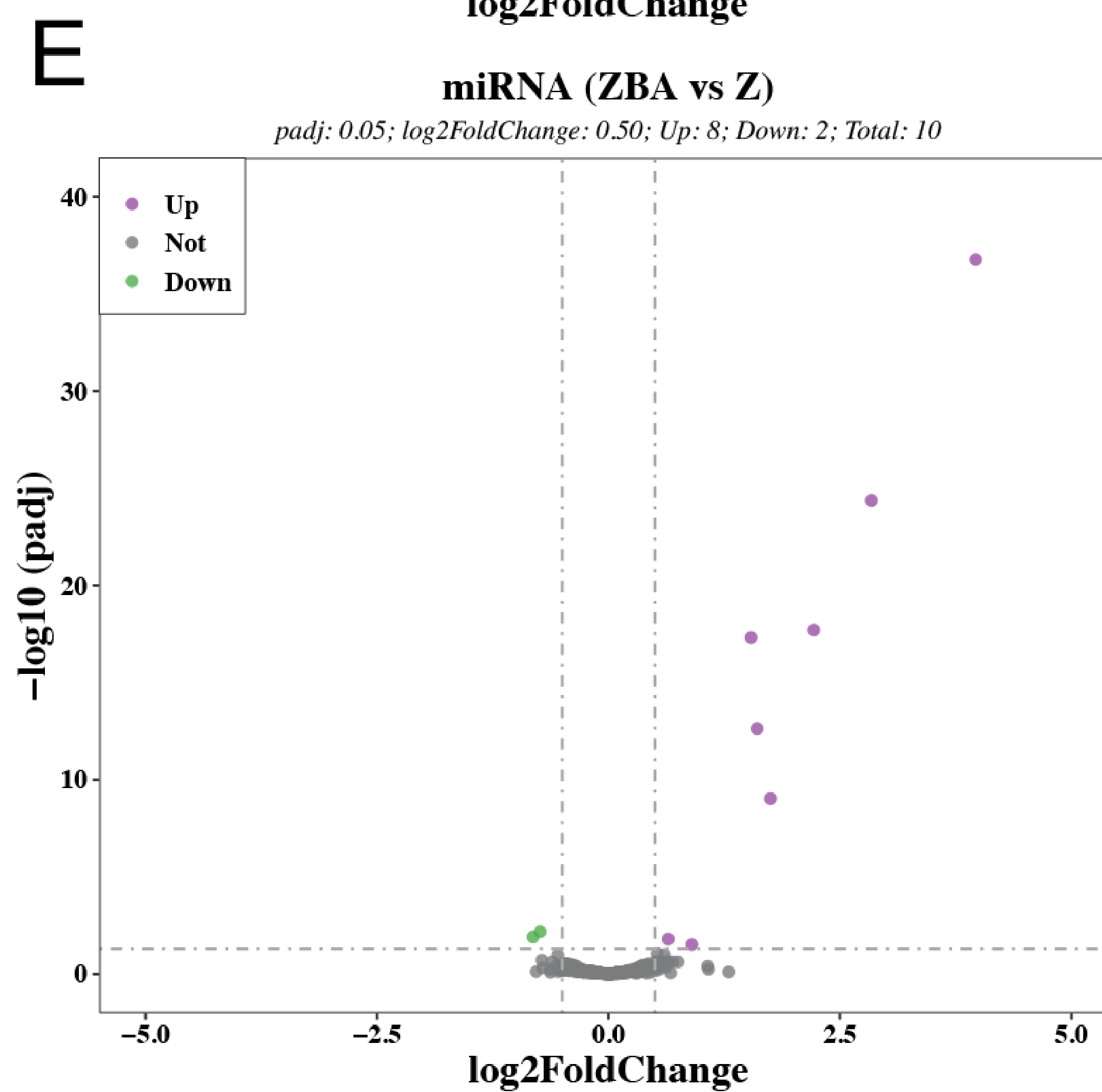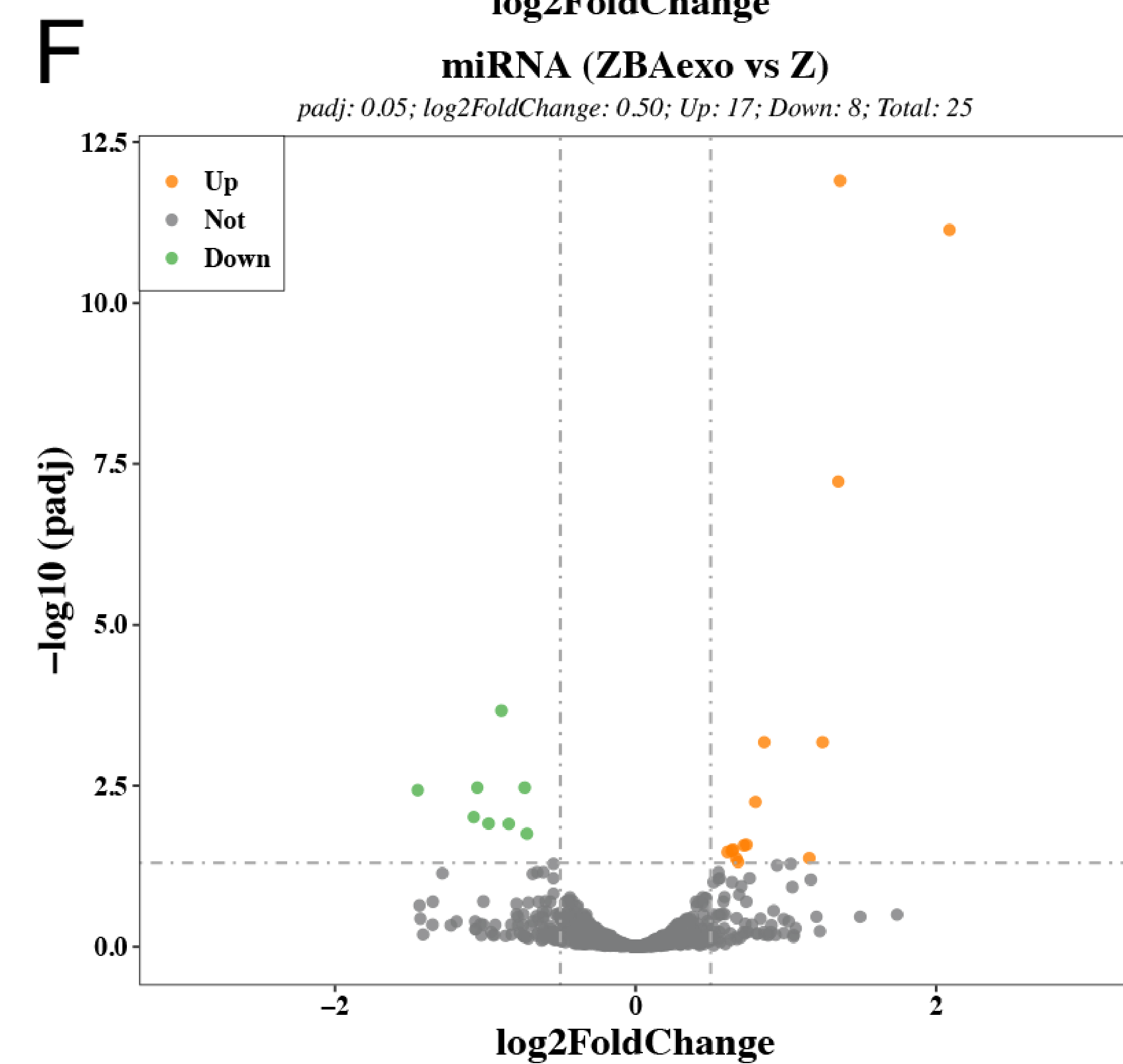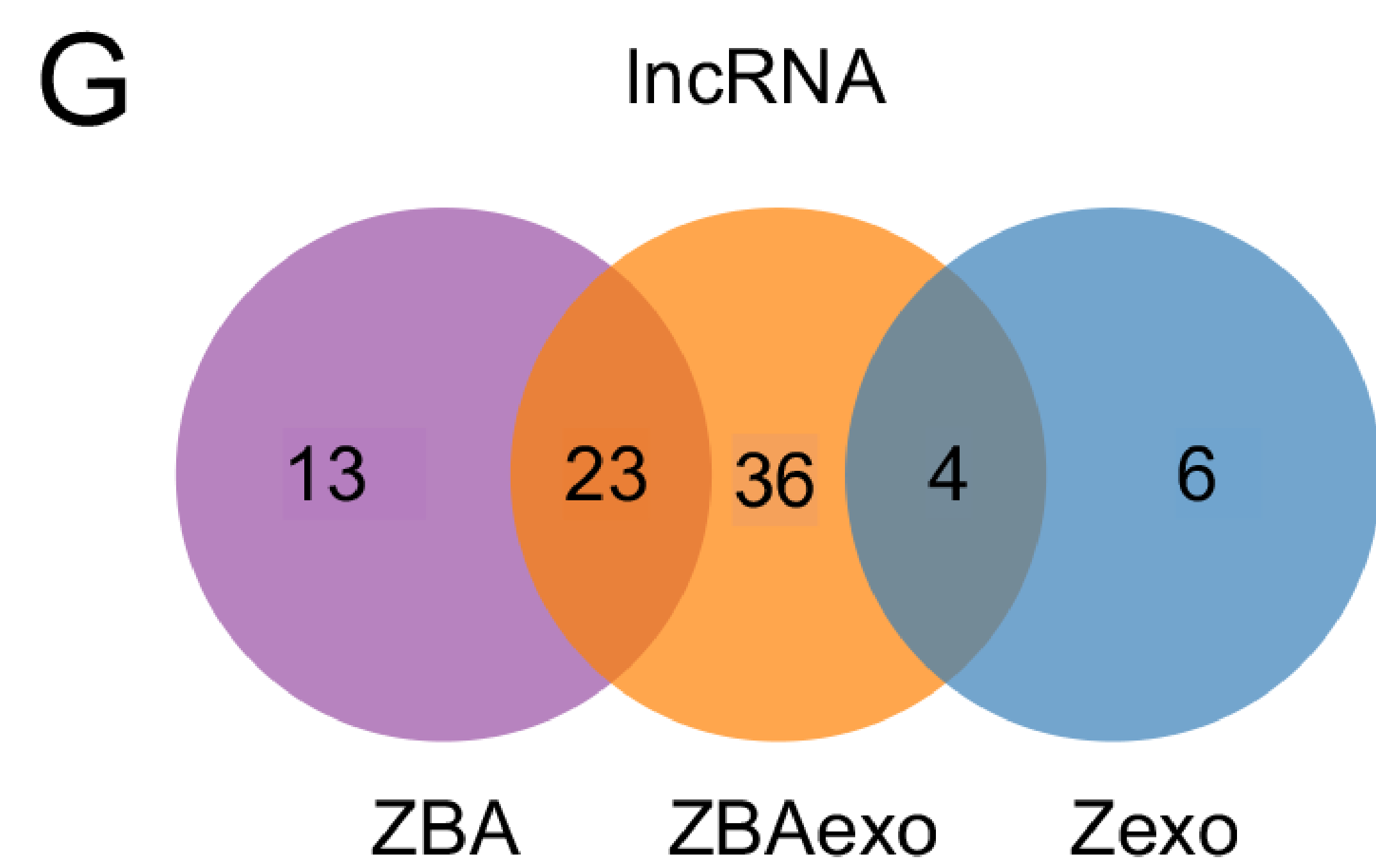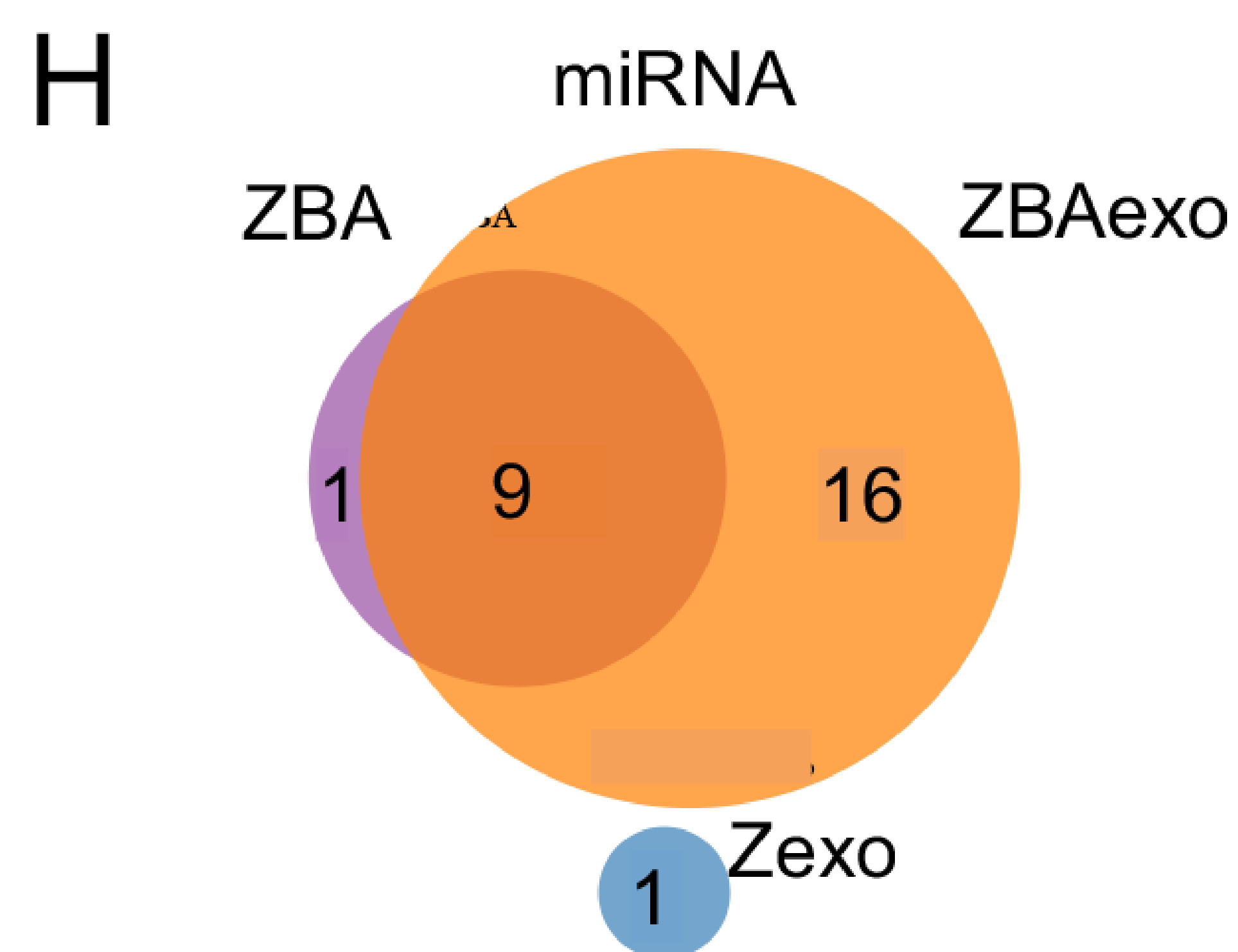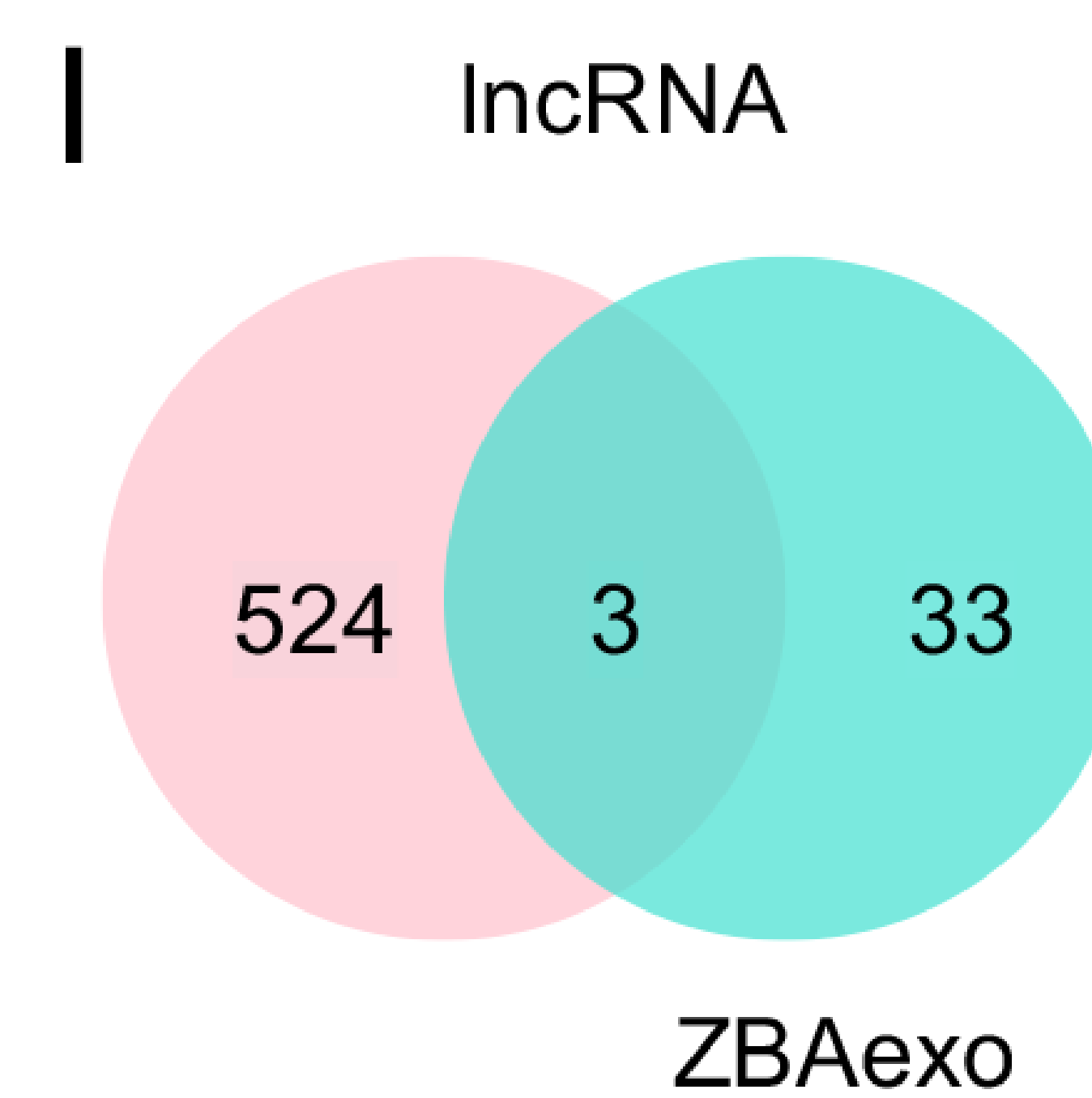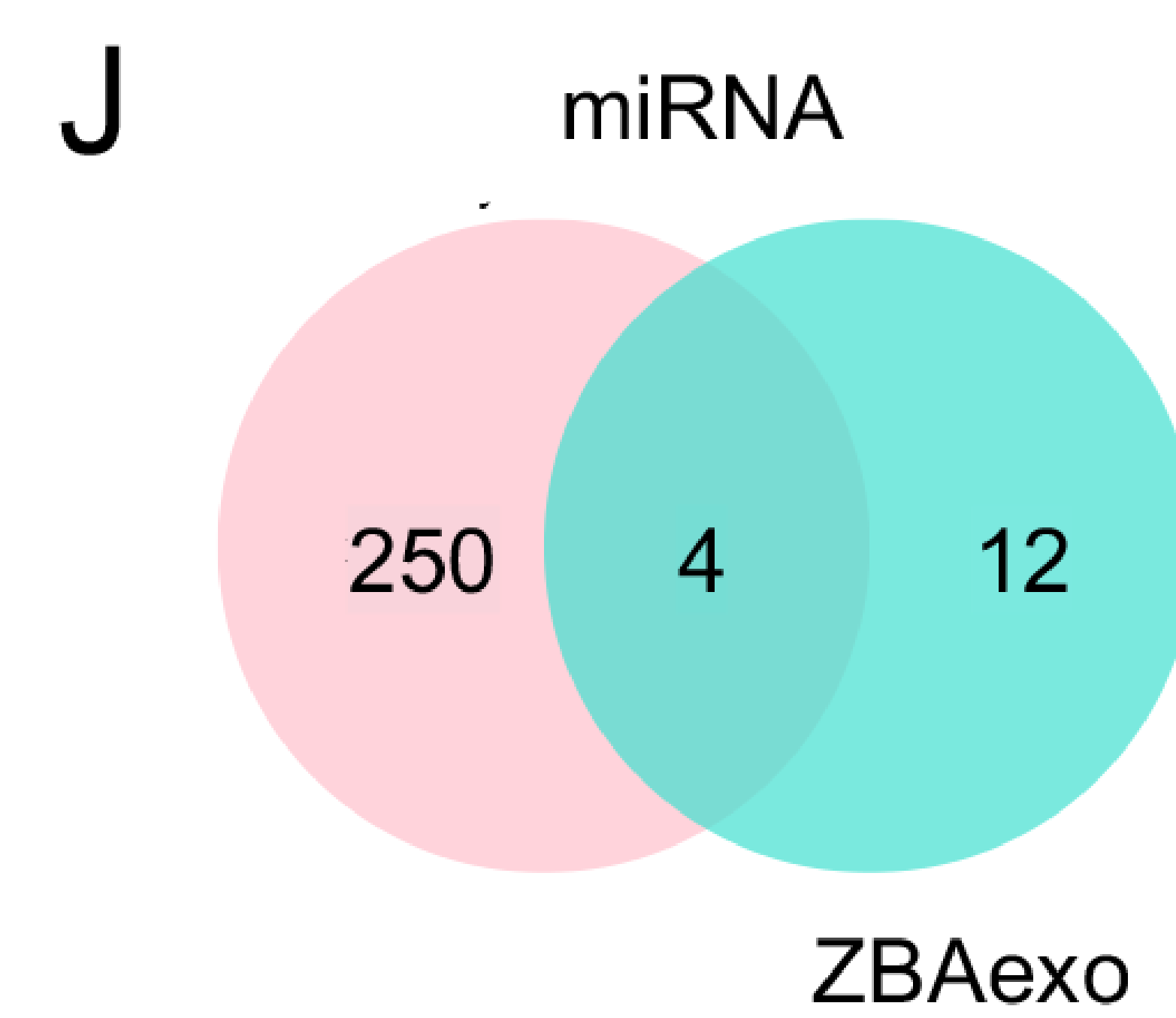

Supplement: Supplemental Information 6 [file peerj-13-20371-s006.pdf]
