# Supplementary material for: Exosome and BCR-ABL mediated molecular alterations in endothelial cells in chronic myeloid leukemia: identification of seven genes and their regulatory network
Source: PeerJ. 2025 Dec 17;13:e20371. doi: 10.7717/peerj.20371 (PMC12717845; doi:10.7717/peerj.20371)
Supplement: Supplemental Information 8 [file peerj-13-20371-s008.pdf]

Supplement table 1

The miRNAs and lncRNAs that interact with GRM1, CDC25C, and SV2A.

mRNA	miRNA	lncRNA
GRM1	hsa-miR-126-5p	AGAP11
CDC25C	hsa-miR-16-5p	AC008124.1
CDC25C	hsa-miR-16-5p	AC064799.2
SV2A	hsa-miR-16-5p	AC008124.1
SV2A	hsa-miR-16-5p	AC064799.2

Supplement table 2

Prediction of small molecule targeted drugs for the genes CAMK2B, CDC25C, SV2A, and GRM1.

gene	drug	sources
CAMK2B	TRIFLUOPERAZINE	NCI
CAMK2B	LINIFANIB	DTC
CAMK2B	SOTRASTAURIN	DTC
CAMK2B	CENISERTIB	DTC
CAMK2B	TAE-684	DTC
CAMK2B	CYC-116	DTC
CAMK2B	SORAFENIB	DTC
CAMK2B	CEDIRANIB	DTC
CAMK2B	LY-2090314	DTC
CAMK2B	SP-600125	DTC
CAMK2B	ILORASERTIB	DTC
CAMK2B	SNS-314	DTC
CAMK2B	IONOMYCIN	NCI
CAMK2B	CAFFEINE	NCI
CAMK2B	NIFEDIPINE	NCI
CAMK2B	ALCOHOL	NCI
CAMK2B	RIMACALIB	TTD
CAMK2B	GSK-269962A	DTC
CAMK2B	VASOPRESSIN	NCI
CDC25C	WITHAFERIN A	DTC
CDC25C	CISPLATIN	NCI
CDC25C	QUERCETIN	NCI
CDC25C	FLUOROURACIL	NCI
SV2A	LEVETIRACETAM	TdgClinicalTrial|ChemblInteractions|TTD
SV2A	BRIVARACETAM	TdgClinicalTrial|ChemblInteractions|TTD
SV2A	SELETRACETAM	TdgClinicalTrial
SV2A	INSULIN	NCI
GRM1	PHILANTHOTOXIN 343	DTC
GRM1	CAFFEINE	NCI
GRM1	INOSITOL	NCI
GRM1	MORPHINE	NCI
