# Supplementary material for: Exosome and BCR-ABL mediated molecular alterations in endothelial cells in chronic myeloid leukemia: identification of seven genes and their regulatory network
Source: PeerJ. 2025 Dec 17;13:e20371. doi: 10.7717/peerj.20371 (PMC12717845; doi:10.7717/peerj.20371)
Supplement: Supplemental Information 9 [file peerj-13-20371-s009.docx]

**Supplement file 1**

**Quantitative Real-Time PCR (qPCR) Method**

1. **RNA extraction**

The collected cells were washed three times with 1 mL of PBS. Then, 1 mL of Trizol was added, and the cells were homogenized until the mixture was no longer viscous. Subsequently, the total RNA was extracted according to the instructions of the Qiagen RNA extraction kit. During elution, 40 μL of RNase-free H₂O was used for each sample.

1. **Removal of Genomic DNA**

Reaction system as shown in Table 1. Reaction conditions: 42°C, 2 min; 4°C storage.

Nucleic acid quantification was performed using a UV-Vis Spectrophotometer (FC-3100, Hangzhou Suizhen Biotechnology Co., Ltd.) at 260 nm, with samples diluted to an optimal concentration range (10 - 100 ng/μL) and measured against RNase/DNase-free water as a blank.

Table 1 Genomic DNA Removal System

| **Component** | **Volume** |
| --- | --- |
| RNA (<1ug) | 8ul |
| 5×gDNA wiper Mix | 2ul |

1. **Reverse Transcription**

HiScript® III 1st Strand cDNA Synthesis Kit (Vazyme Biotech Co., Ltd. Cat: R312-01)

Reaction conditions: 37°C, 15 min; 85°C, 5 s; 4°C storage.

Reaction system as shown in Table 2.

Table 2 Reverse Transcription System

| **Component** | **Volume** |
| --- | --- |
| DNA-free RNA solution | 10ul |
| 10×RT Mix | 2ul |
| HiScript III Enzyme Mix | 2ul |
| Oligo (dT)20um | 1ul |
| Random hexamers | 1ul |
| RNase Free dH2O | 4ul |

1. **qPCR Detection**

The qRT-PCR analysis was performed using the UltraSYBR Mixture (Cat. No. CW2601) from Kangwei Century Biotech Co., Ltd., on the Thermo Fisher Scientific 7500 Real-Time PCR System, with three biological replicates conducted to ensure data reliability and reproducibility. GAPDH was used as an internal reference for qPCR detection of RNA. Primers are shown in Table 3. The Reaction system as shown in Table 4. Reaction conditions: 95°C 10 min; 95°C 10 s, 60°C 30 s (45 cycles).

Table 3 Primers

| **Gene** | **Sequence (5’-3’)** |
| --- | --- |
| BCR-ABL-R | GGGAGCAGCAGAAGAAGTGT |
| BCR-ABL-F | CGGAATGCTGTGGACAGTCT |
| GAPDH-R | GACAAGCTTCCCGTTCTCAG |
| GAPDH-F | GAGTCAACGGATTTGGTCGT |

Table 4 qPCR System

| **Component** | **Volume** |
| --- | --- |
| 2×SYBR green | 10 μL |
| Primer F (10uM) | 0.4 μL |
| Primer R (10uM) | 0.4μL |
| cDNA template | 2μL |
| RNAase Free-H2O | 7.2 μL |

1. Data analysis
